# Evaluation of Environmental Factor Effects on the Polyphenol and Flavonoid Content in the Leaves of *Chrysanthemum indicum* L. and Its Habitat Suitability Prediction Mapping

**DOI:** 10.3390/molecules29050927

**Published:** 2024-02-20

**Authors:** Rei Uranishi, Raju Aedla, Doaa H. M. Alsaadi, Dongxing Wang, Ken Kusakari, Hirotaka Osaki, Koji Sugimura, Takashi Watanabe

**Affiliations:** 1 Department of Medicinal Plant, Graduate School of Pharmaceutical Sciences, Kumamoto University, No. 5-1, Oe-Honmachi, Chuo-ku, Kumamoto 862-0973, Japan; reisamu11@gmail.com (R.U.); doaahushammajeedalsaadi@gmail.com (D.H.M.A.); 227y2009@st.kumamoto-u.ac.jp (D.W.); kusaken@kumamoto-u.ac.jp (K.K.); flyingb69ak@gmail.com (H.O.); sugimura@kumamoto-u.ac.jp (K.S.); 2 BVRIT HYDERABAD College of Engineering for Women, Nizampet Rd, Hyderabad 500090, Telangana, India; 3 Global Center for Natural Resources Sciences, Kumamoto University, No. 5-1, Oe Honmachi, Chuo-ku, Kumamoto 862-0973, Japan

**Keywords:** *Chrysanthemum indicum*, antioxidant, chlorogenic acid, 1,5-dicaffeoylquinic acid, 3,5-dicaffeoylquinic acid, geographic information system, MaxEnt

## Abstract

The leaves of *Chrysanthemum indicum* L. are known to have various bioactive compounds; however, industrial use is extremely limited. To overcome this situation by producing high-quality leaves with high bioactive content, this study examined the environmental factors affecting the phytochemical content and antioxidant activity using *C. indicum* leaves collected from 22 sites in Kochi Prefecture, Japan. Total phenolic and flavonoid content in the dry leaves ranged between 15.0 and 64.1 (mg gallic acid g^−1^) and 2.3 and 11.4 (mg quercetin g^−1^), while the antioxidant activity (EC_50_) of the 50% ethanol extracts ranged between 28.0 and 123.2 (µg mL^−1^) in 1,1-Diphenyl-2-picrylhydrazyl radical scavenging assay. Among the identified compounds, chlorogenic acid and 1,5-dicaffeoylquinic acid were the main constituents in *C. indicum* leaves. The antioxidant activity demonstrated a positive correlation with 1,5-dicaffeoylquinic acid (R^2^ = 0.62) and 3,5-dicaffeoylquinic acid (R^2^ = 0.77). The content of chlorogenic acid and dicaffeoylquinic acid isomers varied significantly according to the effects of exchangeable magnesium, cation exchange capacity, annual temperature, and precipitation, based on analysis of variance. The habitat suitability map using the geographical information system and the MaxEnt model predicted very high and high regions, comprising 3.2% and 10.1% of the total area, respectively. These findings could be used in future cultivation to produce high-quality leaves of *C. indicum*.

## 1. Introduction

*Chrysanthemum* species have been widely used for a long time as food, beverages, cosmetics, and medicines in Asian countries [1]. The flowers are a part of Chinese, Korean, and Japanese traditional medicines used to treat various conditions, such as inflammation, infection, fever, headache, and eye fatigue [1,2]. Among the genus *Chrysanthemum*, only two species, *C. indicum* L. and *C. morifolium* Ramat. are listed in the Japanese Pharmacopoeia [3]. Many studies have investigated the therapeutic potential of these flowers as antioxidant [4], anti-inflammatory [5], hepatoprotective [6], antidiabetic [7], neuroprotective [8], tyrosinase inhibitory [9], and anti-allergic agents [10]. These activities were attributed to the abundance of polyphenols and flavonoids [11,12].

Polyphenols, such as chlorogenic acid and dicaffeoylquinic acid isomers and flavonoids, such as the glycosides of luteolin, apigenin and acacetin are of great dietary importance in *Chrysanthemum* flowers. Sesquiterpenoids, with their various pharmacological activity, are also considered to be important constituents in *Chrysanthemum* flowers [13]. Chlorogenic acid has various activities, such as anti-inflammatory [14], antioxidant [4], and antidiabetic activities [15]. Similarly, dicaffeoylquinic acid isomers exhibit antioxidant [4] and anti-inflammatory [16]. Luteolin glucoside exhibits cardioprotective [17] and anti-inflammatory activities [18]. Likewise, apigenin glucoside has antifungal [19] and anti-inflammatory activities [20]. Acacetin derivatives exhibit cardioprotective [21] and anticancer effects [22].

Regarding the leaves of *Chrysanthemum* species, previous studies have reported that *Chrysanthemum* leaves exhibit histamine release inhibition [10], antimicrobial, anti-inflammatory, and antioxidant activities [23,24,25]. Recently, *C. morifolium* leaves were reported to contain chlorogenic acid, 3,5-dicaffeoylquinic acid, luteoloside, and quercetin [26]. These studies confirmed that *Chrysanthemum* leaves have medicinal value.

Despite the pharmacological interest in the leaves, industrial use is extremely limited. Producing high-quality *Chrysanthemum* leaves with high phytochemical content would be an effective approach for the wider use of the leaves in various fields such as food, beverages, cosmetics, and medicines. This study, therefore, examined environmental factors affecting the phytochemical content and antioxidant activity of *C. indicum* leaves.

*C. indicum* is widely distributed in western Japan (Kyushu, Shikoku, and western Honshu) and grows under various environmental conditions that may significantly affect the accumulation of secondary metabolites [27]. For instance, previous studies have shown that water stress enhances the phenolic content of *C. indicum* [28]. Furthermore, the antioxidant capacity of *C. indicum* was reported to be affected by light quality [29]. Statistical analysis and geographic information systems (GIS) are useful for examining various environmental factors [27].

GIS provides efficient statistical and spatial modeling approaches for preparing habitat suitability maps (HSMs) by integrating several environmental datasets. HSMs define the relationships between species and their environments by understanding their geographical distributions. These spatial models involve statistical analyses to simplify the probability of species existence based on identified correlations between various environmental conditions and the presence or absence of species [30]. The MaxEnt model, utilizing presence occurrence data and employing stochastic generation for associated points, proficiently explains the maximum entropy distribution [31]. Its efficacy extends notably to presence-only datasets featuring constrained sample sizes, demonstrating robust performance even under conditions of data incompleteness [32].

In this study, *C. indicum* leaves were collected from 22 sites in Kochi Prefecture, one of the natural habitats of *C. indicum*. Total phenolic and flavonoid content, antioxidant activity, and the chemical composition were analyzed. Topographical, soil, environmental, and climatic factors were recorded and used for statistical analyses and GIS studies. The HSM of the plant in Kochi was created to predict locations with a high probability of plant growth.

## 2. Results and Discussion

### 2.1. Soil Analysis 

Soil samples collected from the study area showed differences in macro- and micronutrient contents and other soil properties (Table S1). The soil in the study area was a mixture of gravel, coarse sand, and rocks. The soil was slightly acidic, with a pH range of 5.2–6.8. It was rich in ammonium nitrogen (1.7–7.8 mg (100 g)^−1^), nitrate nitrogen (0.1–4.6 mg (100 g)^−1^), phosphorus (5–46 mg (100 g)^−1^), exchangeable potassium (11–54 mg (100 g)^−1^), calcium (39–1230 mg (100 g)^−1^), and magnesium (10–514 mg (100 g)^−1^). The pH (5.2–6.8), soil moisture sensor SM150T output (0.11–0.51 V), and soil bearing capacity (3.6–16.8 t sf^−1^) were measured during the field investigation. The soil moisture (8.0–36.2% vol) was calculated from the SM150T output.

### 2.2. The Phytochemical Content and Antioxidant Activity

**Figure 1:**
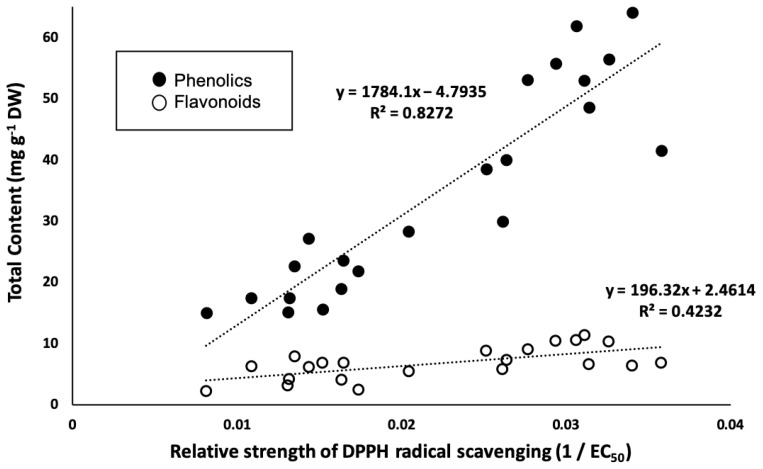
Linear relationship between relative strength of DPPH radical scavenging (1/EC_50_) and the total phenolic and flavonoid content.

Total phenolic and flavonoid content and 1,1-Diphenyl-2-picrylhydrazyl (DPPH) radical scavenging activity are shown in Table 1. Although these measurements demonstrated large variation, more than half of samples resulted in very high antioxidant activity (EC_50_ < 50 µg mL^−1^). These results indicated that *C. indicum* leaves have potent antioxidant activity. The relative strength of DPPH radical scavenging (1/EC_50_) correlated with both total phenolic content (R^2^ = 0.83) and total flavonoid content (R^2^ = 0.42), as shown in Figure 1. This result is consistent with that of a previous study that showed a correlation between antioxidant activity and phytochemical content [4].

**Figure 2:**
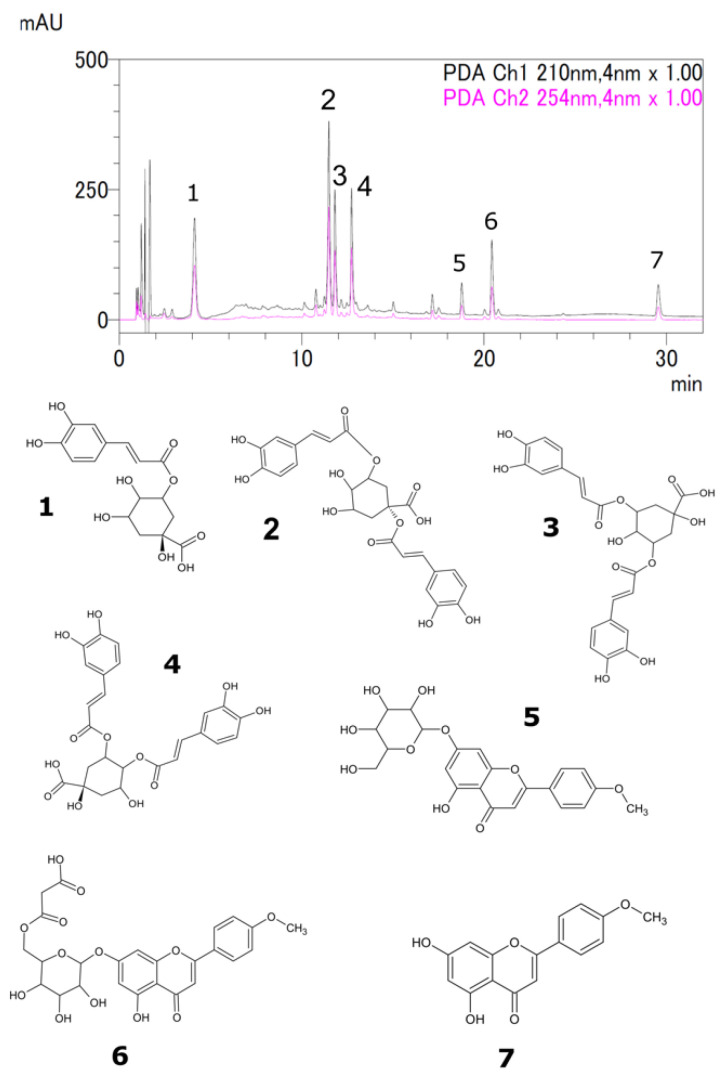
Typical HPLC chromatogram of 50% ethanol extract of *C. indicum* leaves showing peaks of chlorogenic acid (**1**), 1,5-dicaffeoylquinic acid (**2**), 3,5-dicaffeoylquinic acid (**3**), 4,5-dicaffeoylquinic acid (**4**), acacetin 7-*O*-glucoside (**5**), acacetin 7-*O*-malonylglucoside (**6**), and acacetin (**7**).

**Figure 3:**
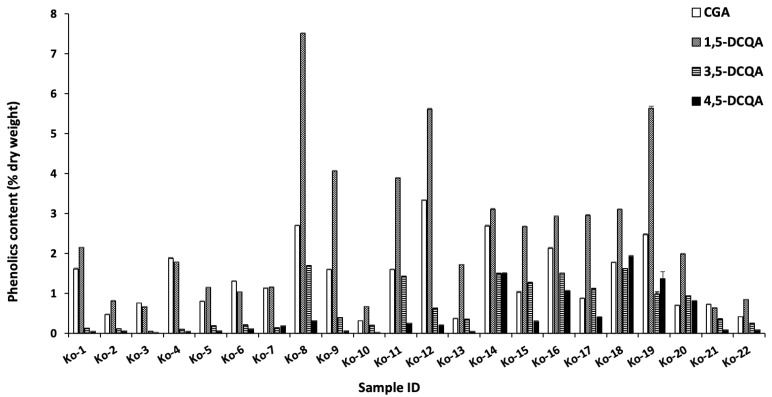
Phenolic content of chlorogenic acid, 1,5-dicaffeoylquinic acid, 3,5-dicaffeoylquinic acid, and 4,5-dicaffeoylquinic acid in the collected leaves.

**Figure 4:**
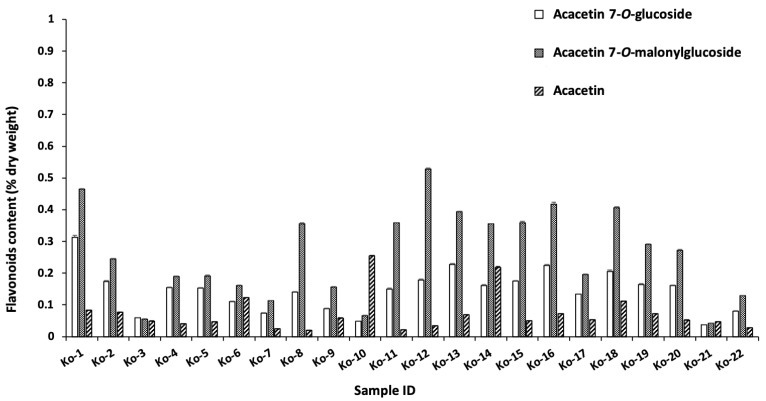
Flavonoid content of acacetin and its glucoside in the collected leaves.

Liquid chromatography-mass spectrometry (LC-MS) and high-performance liquid chromatography (HPLC) were used to analyze the samples [33,34]. The seven major peaks identified were chlorogenic acid, 1,5-dicaffeoylquinic acid, 3,5-dicaffeoylquinic acid, 4,5-dicaffeoylquinic acid, acacetin 7-*O*-glucoside, acacetin 7-*O*-malonylglucoside, and acacetin (Figure 2 and Table 2). The identified compounds were quantified by HPLC, and the results are summarized in Figure 3 and Figure 4. Chlorogenic acid and 1,5-dicaffeoylquinic acid contents varied from 0.3 to 3.3 and 0.6 to 7.5 (% dry weight), and were considered the main phenolic compounds in the *C. indicum* leaf extract. *C. indicum* leaves had little or no flavonoid compounds except for acacetin and the derivatives, whereas *C. indicum* flower contains various flavonoids including luteolin, apigenin, and acacetin, mainly in their glycoside forms [9]. These results suggest that the composition of *C. indicum* leaves chemically differs from its flowers.

Total phenolic and flavonoid content in a flower sample purchased from the market were 12.3 ± 1.0 (mg gallic acid g^−1^ DW) and 9.6 ± 0.0 (mg quercetin g^−1^ DW), respectively, while the antioxidant activity of the 50% ethanol extracts of the flower sample was low (EC_50_ > 125 µg mL^−1^). All samples of *C. indicum* leaves showed higher total phenolic content and antioxidant activity than the flower sample. Therefore, *C. indicum* leaves have great potential as a material with high polyphenol content and high antioxidant activities.

Among the identified compounds, chlorogenic acid, 1,5-dicaffeoylquinic acid, 3,5-dicaffeoylquinic acid, and 4,5-dicaffeoylquinic acid have been reported to exhibit high antioxidant activity owing to the presence of a catechol structure [4]. Catechols have two hydroxyl groups at the ortho-position and can easily donate hydrogen atoms to radical substances to form *o*-quinones [35].

**Figure 5:**
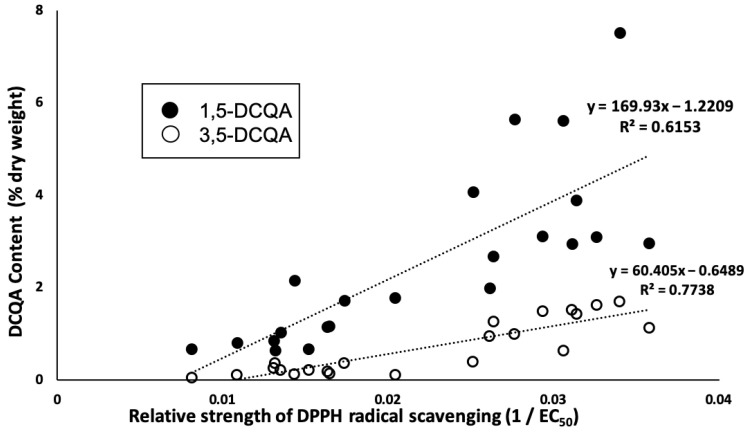
Relationship between relative strength of DPPH radical scavenging (1/EC_50_) and dicaffeoylquinic acid isomers (1,5-dicaffeoylquinic acid and 3,5-dicaffeoylquinic acid).

Statistical analysis using linear correlation showed that the antioxidant activity of the sample was correlated with 1,5-dicaffeoylquinic acid (R^2^ = 0.62) and 3,5-dicaffeoylquinic acid (R^2^ = 0.77), as shown in Figure 5, which is in agreement with a previous study that found that dicaffeoylquinic acid isomers in *C. morifolium* greatly contribute to antioxidant activity [4]. 

Additionally, the variations in the content of the identified antioxidant compounds were explained by the variations in exchangeable magnesium, cation exchangeable capacity, annual precipitation (BIO12), pH, annual mean temperature, precipitation, annual mean temperature (BIO01), clay, bulk density, and topographic wetness index (TWI) based on analysis of variance (ANOVA), as shown in Table 3. Magnesium deficiency in plants can cause a decrease in total phenolic content [36], and exchangeable magnesium is crucial for generating the main phenolic compounds responsible for the antioxidant activity of *C. indicum*. From this result, cation exchange capacity, which shows the ability of soil to hold and supply nutrients such as magnesium and calcium, is also an important parameter for plant growth, as mentioned in previous studies [37,38]. Moreover, this result suggests the significance of climatic factors, such as temperature and precipitation, on the phenolic content of *C. indicum*, as a previous study reported that temperature and precipitation influenced the secondary metabolite content [39].

### 2.3. Habitat Suitability Map (HSM) of MaxEnt Model

**Figure 6:**
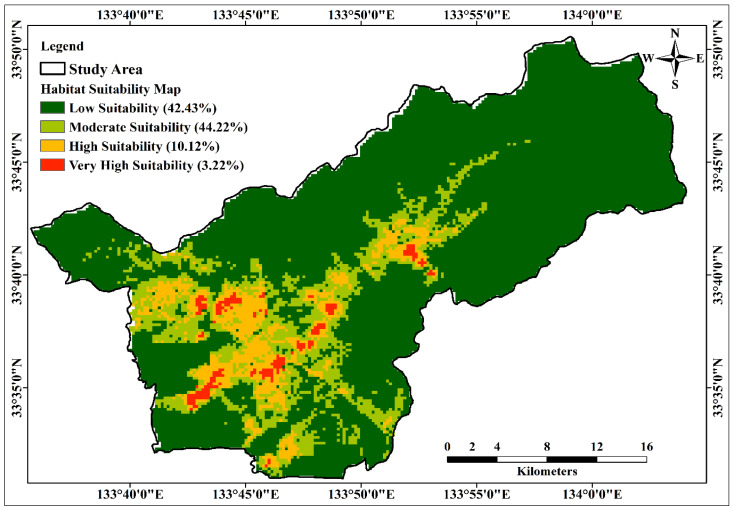
Habitat suitability map of *C. indicum* using MaxEnt model.

The construction of the Habitat Suitability Model (HSM) utilized the MaxEnt model, integrating 32 environmental layers as conditioning factors. These factors included a comprehensive range of topographical, soil, environmental, and climatic variables. The resulting model output was classified into four suitability classes—namely, low, moderate, high, and very high—employing the natural break classification technique within the ArcGIS platform, as shown in Figure 6.

The HSM employs statistical approaches to discriminate optimal plant zones by establishing correlations between conditioning factors and the occurrence of species within the study area. The assessment of the relative importance of variables influencing these conditioning factors is conducted through the rigorous jackknife variance estimation process, specifically applied to quantify the area under the curve (AUC). The outcomes of this analysis are depicted in Figure S1.

In the study area, conditioning factors such as soil sand content, TWI, hillshade, aspect, slope, and precipitation in the driest month (BIO14) had little impact on habitat suitability. In contrast, temperature, precipitation in the wettest month (BIO13) and BIO12, pH in H_2_O, organic content density (OCD), and digital elevation model (DEM) were the most important factors in predicting suitable areas for *C. indicum* growth in the study area. The samples were collected during the water-rich summer season; therefore, precipitation did not substantially affect habitat estimation, although temperature had a significant effect on suitability estimation.

The study area covered an elevation range of 56 to 325 m above sea level, where the DEM and elevation were identified as key factors influencing habitat suitability for *C. indicum*. Particularly, despite considering DEM-derived topographical factors, their impact on predicting habitat suitability for *C. indicum* was relatively modest or inconspicuous. This underscores the intricate relationship between topographical variables and the habitat preferences of *C. indicum* within the studied altitude range. Among the soil factors, the OCD and pH in H_2_O had important effects on the prediction of suitability for *C. indicum*. EC, soil bearing capacity, and pH influenced the suitability estimation, followed by soil bulk density, CEC, SM150T output (soil moisture sensor), and organic carbon content (OCC). 

**Figure 7:**
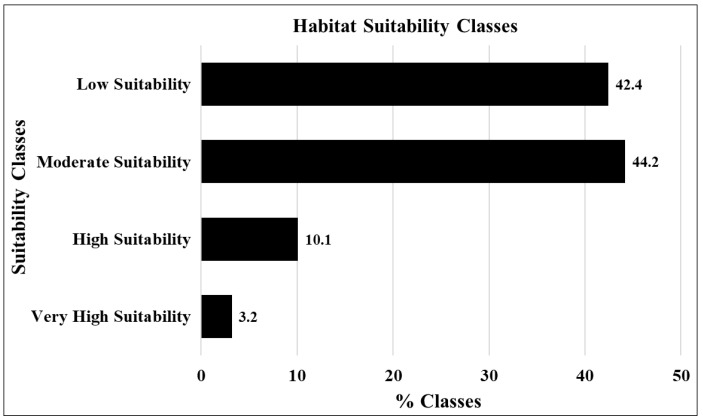
Percentages of habitat suitability classes.

In accordance with the HSM, areas classified as very highly and highly suitable covered 3.2% and 10.1% of the total study area, respectively. The moderately suitable zone comprised a substantial proportion, accounting for 44.2% of the total area, while the low suitability zone encompassed 42.4% of the entire study area, as demonstrated in Figure 7.

From the HSM, all collected samples belonged to high and very high suitability zones, indicating that the study area had good growth conditions for *C. indicum*. This suitability model assists in plant cultivation and preserves habitats with existing plants in the study area.

### 2.4. Validation of MaxEnt Model

**Figure 8:**
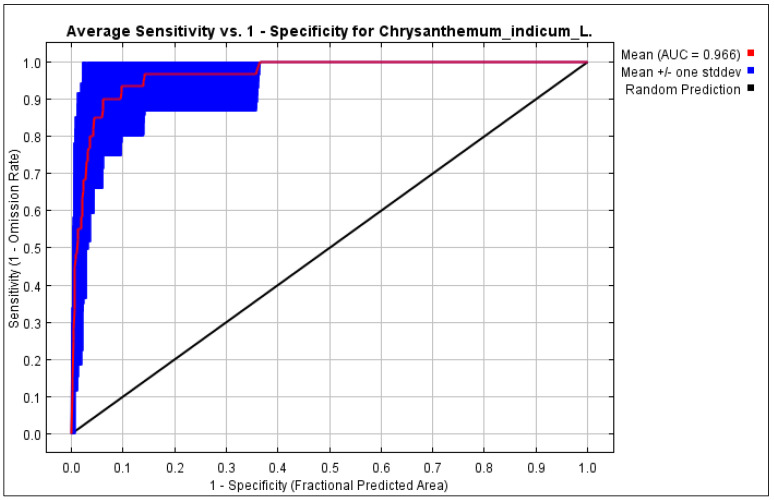
Receiver operator characteristic (ROC) curve with AUC value of *C. indicum* habitat for MaxEnt model.

The validation of the HSM for *C. indicum* involved a comprehensive analysis using a receiver operating characteristic (ROC) curve within the MaxEnt modeling framework. Model performance was assessed through the application of the area under the curve (AUC) metric, as shown in Figure 8. The validation results showed a significant AUC value of 96.6% for the MaxEnt model within the study area. Models with AUC > 0.5 were indicative of superior performance, and in this instance, the MaxEnt model exhibited both logical and acceptable AUC values. The model demonstrated commendable precision in predicting the habitat suitability for *C. indicum* within the selected study area.

## 3. Materials and Methods

### 3.1. Study Area

**Figure 9:**
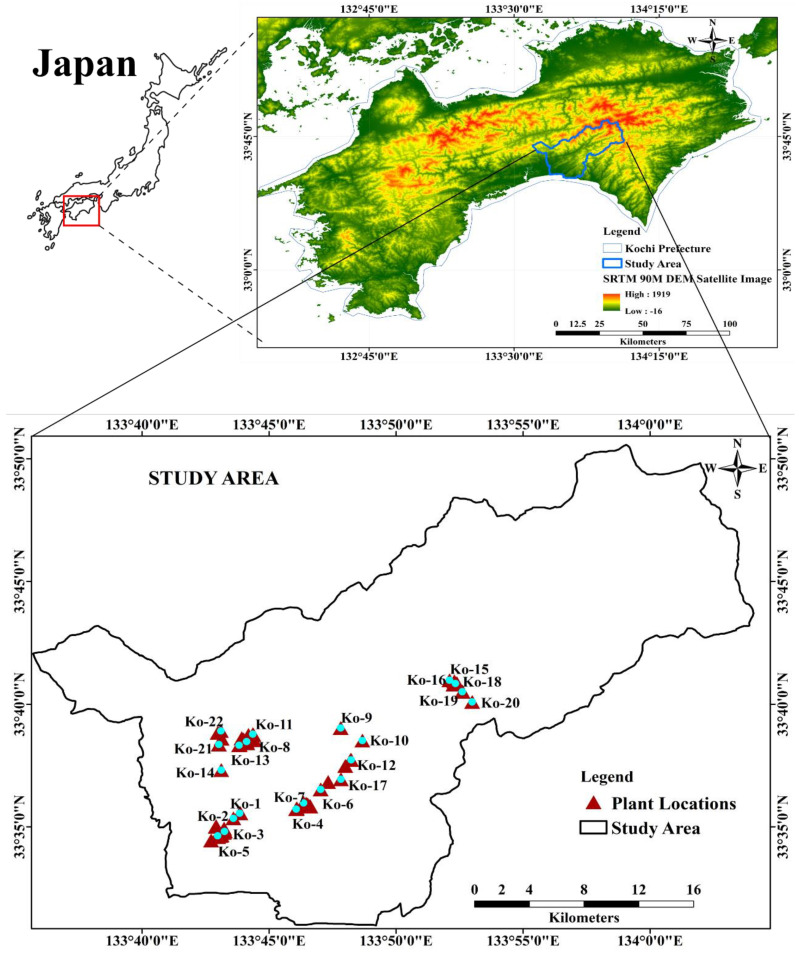
Location map of the study area with the 22 collected plant samples. Kochi Prefecture of Japan shown on ALOS PALSAR 90 M DEM satellite image (https://search.asf.alaska.edu/#/ (accessed on 17 July 2022)).

In this study, the southeastern part of Kochi Prefecture was selected to investigate the natural habitats of *C. indicum*, as shown in Figure 9. Mountains and plains cover this area, with long hours of sunshine and a mild climate throughout the year. The region has a warm and temperate humid subtropical climate with an annual rainfall of approximately 2316 mm. Rainfall was lowest in December (approximately 57 mm) and highest in June (approximately 316 mm). The annual average temperature is 16.4 °C. The highest temperature of 27.2 °C was in August, and the lowest of 5.9 °C was in January, according to the Meteorological Agency of Japan (2020) [40]. 

The study area, covering 702.4 km^2^, is geographically situated between 133°35′0″ and 134°5′0″ E longitude and 33°30′0″ and 33°50′0″ N latitude within the Kochi Prefecture. As illustrated in Figure 9, the geographical delineation is represented by the ALOS PALSAR DEM satellite image and was processed using ArcGIS version 10.5.1 (ESRI Japan Corporation, Tokyo, Japan).

### 3.2. Plant Materials and Data Collection

During field investigations in the study area, 114 locations of *C. indicum* were investigated from 2019 to 2020. Among the investigated locations, 22 were selected for plant collection based on plant maturity, abundance, and status (Figure S2). In June, ten specimens of *C. indicum*, each ranging from 60 to 90 cm in height and exhibiting no signs of diseases, were systematically collected during the fieldwork. Geospatial data, including latitude, longitude, and altitude, were meticulously recorded at the collection sites using a global positioning system (GPS) device (Garmin eTrex 30x from Olathe, KS, USA). The flower of *C. indicum* was purchased from Tochimoto-Tenkaido (Osaka, Japan). A comparison was made between the flower sample and the collected leaf samples in terms of their chemical content and antioxidative activity. The leaf samples were dried in an air-circulating oven at 50 °C for 3 days. After that, the flower and leaf samples were ground into powder separately for extract preparation.

Geographical and climatic information on collected locations of *C. indicum* are shown in Table 4.

### 3.3. Chemicals

Imtakt CD-C18 was bought from Imtakt (Kyoto, Japan). Folin–Ciocalteu phenol reagent and DPPH were obtained from Sigma-Aldrich (St. Louis, MO, USA). 3,5-dicaffeoylquinic acid was purchased from MedChem Express (Monmouth Junction, NJ, USA). 2-Morpholinoethanesulfonic acid monohydrate (MES) was obtained from Dojin Chemical Research Institute (Kumamoto, Japan). Gallic acid was bought from the Tokyo Chemical Industry (TCI) (Tokyo, Japan). Anhydrous sodium carbonate and aluminum chloride (III) were purchased from Kishida Chemical Co., Ltd. (Osaka, Japan). Quercetin was bought from Funakoshi Co., Ltd. (Tokyo, Japan). Trolox, chlorogenic acid, acetonitrile, and other reagents were purchased from FUJIFILM Wako Pure Chemical Industries (Osaka, Japan).

### 3.4. Instrumentation

The EYELA multi-shaker MMS-type and centrifugal evaporators were obtained from Tokyo Rika Kikai (Tokyo, Japan) and Sakuma Seisakusho Co., Ltd. (Tokyo, Japan). A SpectraMax iD5 microplate reader (Molecular Devices, San Jose, CA, USA) was used for the in vitro assays. A Nexera X2 HPLC/UHPLC system (Shimadzu, Kyoto, Japan) was used for quantitative chromatographic analysis. A Bruker amaZon Speed Ion-Trap Mass Spectrometer (Billerica, MA, USA) was used for chemical identification. For the in situ soil analysis, a soil moisture sensor kit SM150T (Delta-T Devices, Cambridge, UK) and a Yamanaka-type soil hardness tester (Fujiwara Seisakusho, Tokyo, Japan) were used. Soil macronutrients were analyzed using an EW-THA1J soil analyzer (Air Water Biodesign, Osaka, Japan).

### 3.5. Soil Sample Collection and Analysis 

Soil samples were acquired for subsequent chemical analyses. The soil samples were subjected to laboratory drying and subsequent chemical analysis using a soil analyzer (EW-THA1J, Air Water Biodesign Co., Ltd., Osaka, Japan).

The pH and soil bearing capacity values of each plant were collected at depths of 10, 25, 50, 75, and 100 cm, and the mean values were calculated for further analysis. An SM150T sensor was used to measure the dielectric properties of moist soil. The output of the SM150T was recorded in Volts (V). The soil refractive index (√ε) was determined by applying the polynomial Equation (1). The relationship between soil moisture (θ) and √ε was found to be linear (2). In the case of mineral soils, θ was calculated based on the output of the SM150T device using Equation (3) as per manufacturer’s recommendation [41].

√ε = 1.0 + 14.4396V − 31.2587V^2^ + 49.0575V^3^ − 36.5575V^4^ + 10.7117V^5^ (1)

√ε = a_0_ + a_1_ × θ (2)

where a_0_ is the intercept, and a_1_ is the slope (mineral soil: a_0_ = 1.6, a_0_ = 8.4).

θ_mineral_ = −0.0714 + 1.7190V − 3.7213V^2^ + 5.8402V^3^ − 4.3521V^4^ + 1.275V^5^ (3)

### 3.6. Preparation of Plant Extract and Samples

About 1 g of dry plant powder was mixed with 40 mL of 50% ethanol and sonicated at 50 °C for 1 h. Subsequently, it was extracted at 25 °C for 1 d with shaking at 100 rpm. The extract was filtered through filter paper no. 2 (Advantec, Tokyo, Japan) and concentrated using a centrifuge evaporator. Finally, it was dried overnight under high vacuum (<10 Pa). 

The dried extracts were dissolved in dimethyl sulfoxide (DMSO) at 0.5% and used to evaluate DPPH radical scavenging and polyphenolic and flavonoid contents. 

### 3.7. Evaluation of DPPH Radical Scavenging Activity

The DPPH radical scavenging activity of the extracts was determined using the method described by Oki et al. with modifications [42]. Briefly, 100 µL of the dilution series of samples and positive control (Trolox) in 50% ethanol were transferred to the 96-well assay plate with 50 µL of MES buffer (0.2 M, pH 6.0) and 50 µL of DPPH (800 µM). For the negative control experiment, 100 µL of 50% ethanol was used instead of a sample solution. The mixture was allowed to stand in the dark at 25 °C for 20 min, and the absorbance was measured at 520 nm. Measurements were performed in triplicate, and the DPPH radical scavenging activity was calculated using Equation (4):

DPPH radical scavenging activity% = [(A_0_ − A_1_)/A_0_] × 100 (4)

where A_0_ is the absorbance of the negative control and A_1_ refers to the sample absorbance.

The EC_50_ for DPPH radical scavenging was calculated as previously described [43]. 

### 3.8. Total Phenolic Content

The total phenolic content of the extracts was determined using the Folin–Ciocalteu method described by Julkunen-Titto et al. [44]. In a 96-well plate, 20 μL of plant extract and 100 μL of 10% Folin–Ciocalteu’s phenol reagent were added, and the plate was allowed to stand for 5 min. Next, 80 μL of 2.5% sodium bicarbonate solution was added and allowed to stand for 1 h at 25 °C. Absorbance was measured at 755 nm using a microplate reader. The total phenolic content was determined as gallic acid equivalents (mg g^−1^ extract) based on a standard curve constructed with gallic acid.

### 3.9. Total Flavonoid Content

The total flavonoid content in the extracts was determined using the reported method with some changes [45]. In total, 25 μL of plant extract and 75 μL of ethanol were added to a 96-well plate. After that, 5 μL of 10% aluminum chloride (III) and 145 μL of pure water were filled. The 96-well plate was stored in the dark at 25 °C for 30 min. Absorbance was measured at 420 nm using a microplate reader. The total flavonoid content was determined as quercetin equivalent (mg g^−1^ extract) according to the standard curve made with quercetin.

### 3.10. Chromatographic Quantification and Analysis

A 50% ethanol dry extract of *C. indicum* was dissolved in 50% methanol to prepare a 0.5% solution. After centrifugation at 16,000 rpm for 10 min, the supernatant was collected with HPLC using an Imtakt CD-C18 column (2 × 150 mm, 3 µm). The mobile phase comprised 10% acetonitrile containing 0.1% acetic acid (A) and acetonitrile (B). The mixing ratio (A:B) was linearly changed from 100:0 to 53.3:46.7 for 35 min after the start, and then the column was washed with 100% B solution for 9 min. The injection volume was 1.0 µL, flow rate was 0.3 mL min^−1^, column temperature was 40 °C, and PDA detection wavelength was 200−360 nm.

Chlorogenic acid, 3,5-dicaffeoylquinic acid, and 4,5-dicaffeoylquinic acid were dissolved in 50% ethanol at 10, 30, 100, 300, and 1000 µg mL^−1^ to obtain the corresponding HPLC standard curves (R^2^ > 0.99). 1,5-dicaffeoylquinic acid was similarly dissolved in 50% ethanol up to 2000 µg mL^−1^. Acacetin was dissolved in DMSO–water (8:2) to obtain the corresponding HPLC standard curve (R^2^ > 0.99). Acacetin 7-*O*-glucoside and acacetin 7-*O*-malonylglucoside contents were also determined as acacetin equivalents, based on a standard curve of acacetin.

For the LC-MS analysis, 0.1% extract samples were analyzed under the same elution conditions as described above.

### 3.11. MaxEnt Model

MaxEnt software version 3.4.1 was downloaded from https://biodiversityinformatics.amnh.org/open_source/maxent/ (accessed on 21 December 2021) and used to predict habitat suitability for *C. indicum*. 

In this investigation, 70% of the georeferenced dataset pertaining to *C. indicum* species was allocated for training, while the remaining 30% served for model validation. The collected species presence-only data, along with environmental layers in both continuous and categorical formats, were input into the MaxEnt model. The model ran with ten replicates to predict the distribution of *C. indicum*, and the relative importance of conditioning factors was systematically assessed using the jackknife test. The resulting output from the MaxEnt model delineates the potential distribution of *C. indicum* within the study area. Subsequently, the ASCII file (.ASC) was imported into ArcGIS 10.1 software (licensed) to generate a spatial distribution map of *C. indicum*. Higher values on the map signify an elevated likelihood of species presence, while lower values indicate a diminished degree of species adaptation.

#### 3.11.1. Dataset Preparation for Habitat Suitability Modeling

To develop an efficient and suitable species habitat model, various climatic, environmental, topographical, and soil layers in the study area are required [46]. Therefore, 32 factors that influenced the habitat suitability for *C. indicum* were identified in the study area. The selected conditioning factors were classified as topographical, soil, environmental, or climatic. Topographical factors were extracted from an Advanced Land Observing Satellite-Digital Elevation Model (ALOS-DEM) satellite image with a 12.5 × 12.5 spatial resolution. This image was downloaded from the ALOS PALSAR (The Phased Array Type L-band Synthetic Aperture Radar) satellite website (https://search.asf.alaska.edu/#/ (accessed on 17 July 2022)). In addition, soil factors except for SM150T output and soil bearing capacity were downloaded from SoilGrids250m 2.0 (http://soilgrids.org (accessed on 11 May 2022)) at a depth of 0.15 m. Climatic factors, including annual mean rainfall and temperature were obtained from the Japan Meteorological Agency (https://www.jma.go.jp/ (accessed on 5 May 2022)). Additionally, 6 bioclimatic variables out of 19 [47], including BIO01, BIO05, BIO06, BIO12, BIO13, and BIO14, for 2010–2018 were downloaded from the WorldClim website (https://www.worldclim.org/ (accessed on 3 May 2022)). The environmental factors were prepared using topographical maps at a scale of 1:250,000 (Table S2).

Thematic maps of soil factors, climatic data, and environmental layers were prepared using the interpolation method of the inverse distance-weighted (IDW) spatial analysis technique in ArcGIS 10.1 version. All thematic raster layers were converted into the ASCII format with the same spatial resolution. All layers were then imported into the MaxEnt Version 3.4.1 software tool as environmental layers to predict habitat suitability for *C. indicum* species. The thematic maps and various conditioning factors associated with each plant location, used in predicting the habitat suitability of the study area, are illustrated in Figure S3 and detailed in Table S2. Table S3 presents a comprehensive list of conditioning factors, including their categories and data scales.

#### 3.11.2. Validation of HSM

The validation process for the HSM involved employing the ROC curve to assess the predicted HSMs generated by the MaxEnt model within the study area. The AUC served as a metric to gauge the prediction accuracy of the MaxEnt model. Importantly, AUC values ranging from 0.5 to 1 were observed, with higher values indicative of more precise results. Within the ROC method, the *x*-axis portrays the cumulative percentage of suitability classes, while the *y*-axis represents the cumulative percentage of the training set within those classes [48]. It is important to highlight that the ROC method is essential for accurately modeling the estimated distribution of plant species [49].

### 3.12. Statistical Tests

Mean values and standard deviations were calculated for all measurements. The linear correlation between the examined variables, plant activity, and secondary metabolite content was calculated using the Data Analysis function of the Excel Add-in and XLSTAT statistical and data analysis solutions (Addinsoft, 2020, New York, NY, USA). Correlations with a *p* < 0.05 were considered significant.

## 4. Conclusions

In the present study, *C. indicum* leaves were revealed to have a potent antioxidant activity, which was possibly explained by the high content of chlorogenic acid and dicaffeoylquinic acid isomers. The leaves had little or no flavonoid compounds except for acacetin and the derivatives. This is in contrast with the flavonoid composition of the flower part, which was reported to contain various flavonoids including luteolin, apigenin, and acacetin, mainly in their glycosides.

The content of chlorogenic acid and dicaffeoylquinic acid isomers was significantly affected by exchangeable Mg and cation exchange capacity as well as BIO12, pH, annual mean temperature, precipitation, BIO01, clay, bulk density, and TWI. The MaxEnt model provided accurate HSM results for assessing the habitat suitability for *C. indicum*. Overall, climatic factors such as temperature, BIO13, and BIO12 had the strongest effect on habitat suitability. Soil factors including pH in H_2_O and OCD also significantly affected habitat predictions. These findings could be used for the future cultivation and investigation of *C. indicum* to obtain high phenolic content and bioactivity to meet industrial needs.

## Figures and Tables

**Table 1:** Total phenolic and flavonoid content and DPPH radical scavenging activity of 50% ethanol extracts of *C. indicum* leaves *.

ID ^a^	Total Phenolic Content (mg Gallic Acid g^−1^ DW) ^b^	Total Flavonoid Content (mg Quercetin g^−1^ DW) ^b^	EC_50_ for DPPH Radical Scavenging (µg mL^−1^) ^c^
Ko-1	27.2 ± 0.1	6.2 ± 0.1	69.8 ± 1.5
Ko-2	17.5 ± 0.1	6.3 ± 0.6	92.2 ± 1.3
Ko-3	15.0 ± 0.7	2.3 ± 0.0	123.2 ± 3.5
Ko-4	28.3 ± 0.1	5.5 ± 0.0	49.0 ± 3.9
Ko-5	18.9 ± 0.2	4.1 ± 0.1	61.3 ± 10.0
Ko-6	22.7 ± 0.2	8.0 ± 0.0	74.2 ± 2.7
Ko-7	23.6 ± 0.0	6.9 ± 0.0	60.8 ± 6.6
Ko-8	64.1 ± 0.2	6.4 ± 0.1	29.4 ± 4.7
Ko-9	38.6 ± 0.7	8.9 ± 0.1	39.8 ± 4.5
Ko-10	15.6 ± 0.5	6.9 ± 0.2	65.9 ± 1.8
Ko-11	48.6 ± 1.1	6.7 ± 0.0	31.9 ± 0.2
Ko-12	61.9 ± 0.3	10.6 ± 0.1	32.7 ± 0.8
Ko-13	21.9 ± 0.1	2.5 ± 0.2	57.7 ± 1.9
Ko-14	55.8 ± 0.8	10.5 ± 0.0	34.1 ± 0.8
Ko-15	40.1 ± 1.6	7.4 ± 0.1	38.0 ± 0.6
Ko-16	53.0 ± 0.6	11.4 ± 0.1	32.2 ± 3.0
Ko-17	41.5 ± 0.2	6.9 ± 0.1	28.0 ± 1.6
Ko-18	56.4 ± 0.3	10.3 ± 0.1	30.7 ± 4.3
Ko-19	53.1 ± 0.6	9.1 ± 0.0	36.2 ± 7.6
Ko-20	30.0 ± 0.1	5.9 ± 0.1	38.3 ± 1.2
Ko-21	17.5 ± 0.0	4.2 ± 0.0	76.0 ± 1.3
Ko-22	15.2 ± 0.2	3.2 ± 0.0	76.5 ± 2.2

* Data represent the average ± standard deviation from triplicate measurements of a mixed sample collected in the same location. ^a^ ID represents *C. indicum* collection sites, where Ko refers to Kochi Prefecture. ^b^ DW represents dry weight of the leaves sample of *C. indicum.* ^c^ EC_50_ represents effective concentration for 50% scavenging of DPPH radicals.

**Table 2:** Identified compounds in LC-MS.

Peak No.	Identified Compound	Observed Ion (Ion–Trap MS)	Retention Time (min.)
**1**	Chlorogenic acid	*m*/*z* [M + H]^+^: 355.0, *m*/*z* [M − H]^−^: 353.1	4.1
**2**	1,5-dicaffeoylquinic acid	*m*/*z* [M + H]^+^: 517.1, *m*/*z* [M − H]^−^: 515.2	10.7
**3**	3,5-dicaffeoylquinic acid	*m*/*z* [M − H]^−^: 515.1	11.1
**4**	4,5-dicaffeoylquinic acid	*m*/*z* [M + H]^+^: 517.1, *m*/*z* [M − H]^−^: 515.1	12.1
**5**	Acacetin 7-*O*-glucoside	*m*/*z* [M + H]^+^: 447.0, *m*/*z* [M + H − 162]^+^: 285.0	18.4
**6**	Acacetin 7-*O*-malonylglucoside	*m*/*z* [M + H]^+^: 533.1, *m*/*z* [M + H − 248]^+^: 285.0	20.0
**7**	Acacetin	*m*/*z* [M + H]^+^: 285.0, *m*/*z* [M − H]^−^: 283.0	29.2

**Table 3:** ANOVA analysis of variables effects on chlorogenic acid, 1,5-dicaffeoylquinic acid, 3,5-dicaffeoylquinic acid, 4,5-dicaffeoylquinic acid, and acacetin 7-*O*-malonylglucoside.

Variables	CGA (%)		1,5-DCQA (%)		3,5-DCQA (%)		4,5-DCQA (%)		Acacetin 7-*O*-Malonylglucoside (%)	
	F Value	R^2^	F Value	R^2^	F Value	R^2^	F Value	R^2^	F Value	R^2^
Exchangeable magnesium	2.25	0.85	4.12 *	0.91	17.87 ***	0.98	90.23 ***	0.99	1.21	0.75
Cation exchangeable capacity	3.61 *	0.69	5.01 *	0.76	2.98 *	0.65	16.61 ***	0.91	2.05	0.65
BIO12	1.26	0.49	1.31	0.50	2.66	0.67	13.21 ***	0.91	1.09	0.45
pH	0.70	0.48	1.79	0.7	11.48 ***	0.94	2.07	0.73	4.73 *	0.86
Annual mean temperature and precipitation	1.22	0.57	3.11 *	0.77	5.34 *	0.86	9.73 **	0.92	1.47	0.62
BIO01	1	0.53	2.09	0.70	4.74 *	0.84	9.65 **	0.91	0.88	0.49
Clay	0.66	0.17	1.13	0.26	2.91	0.48	9.09 ***	0.74	1.13	0.26
Bulk density	2.21	0.88	4.25	0.93	8.70 *	0.97	4.03	0.93	0.87	0.74
TWI	2.15	0.90	4.20	0.95	7.34 *	0.97	3.04	0.93	4.54	0.95

* *p* < 0.05, ** *p* < 0.01, and *** *p* < 0.001.

**Table 4:** Geographical and climatic information of collection sites of *C. indicum*.

ID ^a^	Longitude (E)	Latitude (N)	Elevation (m)	Mean Annual Temperature (°C) ^b^	Mean Annual Rainfall (mm) ^b^
Ko-1	133°43′50.4″	33°35′33.12″	326	15.9	2059
Ko-2	133°43′35.5″	33°35′20.82″	296	16.3	2054
Ko-3	133°43′14.59″	33°34′48.65″	197	16.5	2055
Ko-4	133°43′14.34″	33°34′48.1″	199	16.5	2055
Ko-5	133°42′58.3″	33°34′38.06″	226	16.5	2055
Ko-6	133°42′59.08″	33°34′35.9″	220	16.5	2055
Ko-7	133°42′57.57″	33°34′38.28″	232	16.5	2055
Ko-8	133°44′6.93″	33°38′28.55″	141	15.7	2017
Ko-9	133°43′55.26″	33°38′36.82″	191	15.3	2042
Ko-10	133°43′59.79″	33°38′33.49″	214	15.2	2045
Ko-11	133°44′21.54″	33°38′46.85″	301	15.2	2045
Ko-12	133°44′11.05″	33°38′44.76″	322	15.2	2045
Ko-13	133°43′49.03″	33°38′19.7″	86	16.1	1995
Ko-14	133°43′6.83″	33°37′18.78″	176	16.6	2000
Ko-15	133°52′20.23″	33°40′50.78″	275	14.3	2049
Ko-16	133°52′23.51″	33°40′50.72″	263	14.3	2049
Ko-17	133°52′18.18″	33°40′5″	273	14.3	2049
Ko-18	133°52′36.35″	33°40′30.73″	292	13.9	2073
Ko-19	133°52′36.33″	33°40′30.54″	293	13.9	2073
Ko-20	133°52′59.64″	33°40′5.54″	296	14.0	2078
Ko-21	133°44′24.09″	33°38′32.58″	97	15.2	2045
Ko-22	133°43′8.01″	33°38′35.53″	75	15.6	2022

^a^ ID represents the location of the *C. indicum* collection, where Ko refers to the Kochi Prefecture. ^b^ Acquired from Japan Meteorological Agency, General Information on Climate of Japan (https://www.jma.go.jp/ (accessed on 5 May 2022)).

## Data Availability

Data are contained within the article and supplementary materials.

## Supplementary Materials

The following supporting information can be downloaded at: https://www.mdpi.com/article/10.3390/molecules29050927/s1. Table S1: Soil data of the plant collection sites. Table S2: Various conditioning factors were used to predict habitat suitability maps using the MaxEnt model. Table S3: Conditioning factors used to predict habitat suitability for *C. indicum*. Figure S1: Analysis of the relative importance of effective environmental variables. Figure S2: Photographs of *Chrysanthemum indium* L. in situ (at the collection sites). Figure S3: Thematic maps for predicting habitat suitability using the MaxEnt model for the study area.
