# Supplementary material for: Evaluation of Environmental Factor Effects on the Polyphenol and Flavonoid Content in the Leaves of Chrysanthemum indicum L. and Its Habitat Suitability Prediction Mapping
Source: Molecules. 2024 Feb 20;29(5):927. doi: 10.3390/molecules29050927 (PMC10934983; doi:10.3390/molecules29050927)
Supplement: Supplementary file 1 [file molecules-29-00927-s001.zip › molecules-2822352-supplementary.pdf]

Evaluation of Environmental Factor Effects on the Polyphenol and Flavonoid Content in the Leaves of *Chrysanthemum indicum* L. and Its Habitat Suitability Prediction Mapping

Rei Uranishi 1, Raju Aedla 2,3,*, Doaa H. M. Alsaadi 1, Dongxing Wang 1, Ken Kusakari 1, Hirotaka Osaki 1, Koji Sugimura 1,3 and Takashi Watanabe 1,3,*

[1] Department of Medicinal Plant, Graduate School of Pharmaceutical Sciences, Kumamoto University, 5-1 Oe-Honmachi, Chuo-ku, Kumamoto, Kumamoto 862-0973, Japan; reisamu11@gmail.com (R.U.); doaahushammajeedalsaadi@gmail.com (D.H.M.A.); 227y2009@st.kumamoto-u.ac.jp (D.W.); kusaken@kumamoto-u.ac.jp (K.K.); flyingb69ak@gmail.com (H.O.); sugimura@kumamoto-u.ac.jp (K.S.)

[2] BVRIT HYDERABAD College of Engineering for Women, Nizampet Rd, Hyderabad 500090, Telangana, India;

[3] Global Center for Natural Resources Sciences, Kumamoto University, No. 5-1, Oe Honmachi, Chuo-ku, Kumamoto 862-0973, Japan

* Correspondence:rajuaedla@kumamoto-u.ac.jp or raju.aedla@bvrithyderabad.edu.in (R.A.); wtakashi@kumamoto-u.ac.jp (T.W.)

Supplementary Material

The following supporting information was included:

Table S1. Soil data of the plant collection sites.

Table S2. Various conditioning factors were used to predict habitat suitability maps using the MaxEnt model.

Table S3. Conditioning factors used to predict habitat suitability for *C. indicum*.

Figure S1. Analysis of the relative importance of effective environmental variables.

Figure S2. Photographs of Chrysanthemum *indium* L. in situ (at the collection sites)

Figure S3. Thematic maps for predicting habitat suitability using the MaxEnt model for the study area.

Table S1. Soil data of the plant collection sites

ID [a]	pH [b]	Soil Bearing Capacity ($t\ sf^{-1}$) [b]	SM150T Output (V) [b]	soil moisture (% vol) [c]	Electrical Conductivity (EC) ($mS\ cm^{-1}$)	Silt (%)	Sand (%)	Clay (%)	pH in H_2O	Organic Carbon Density ($kg\ m^{-3}$)	Organic Carbon Content ($g\ Kg^{-1}$)	Cation Exchange Capacity ($mmolc\ kg^{-1}$)	Bulk Density ($Kg\ m^{-3}$)	Ammonium-nitrogen (NH_3-N) ($mg\ (100\ g)^{-1}$)	Nitrate –nitrogen (NO_3-N) ($mg\ (100\ g)^{-1}$)	Available Phosphorus (P) ($mg\ (100\ g)^{-1}$)	Exchangeable Potassium ($mg\ (100\ g)^{-1}$)	Exchangeable Calcium (Ca^{2+})($mg\ (100\ g)^{-1}$)	Exchangeable Magnesium ($mg\ (100\ g)^{-1}$)	Exchangeable Manganese ($mg\ (1\ kg)^{-1}$)	Available Iron (Fe) ($mg\ (1\ kg)^{-1}$)	Available Copper (Cu) ($mg\ (1\ kg)^{-1}$)	Available Zinc (Zn) ($mg\ (1\ kg)^{-1}$)	Boron (B) ($mg\ (1\ kg)^{-1}$)
Ko-1	6.6	16.8	0.11	7.98	0.14	35	37	28	5.4	289	28	16	1287	1.7	0.83	5	22	816	36	24.7	2.3	0.1	31.6	1.1
Ko-2	5.6	9.8	0.25	20.13	0.46	35	40	25	5.6	293	26	17	1304	7.8	0.1	19	39	1230	30	26.7	8.7	0.4	3.2	2.1
Ko-3	6.7	10.3	0.14	11.07	0.08	35	40	26	5.5	248	25	15	1338	2.3	0.36	5	40	271	39	25.6	9.0	0.2	1.0	0.5
Ko-4	5.3	12	0.39	29.03	0.19	35	40	26	5.5	248	25	15	1338	2.5	0.97	8	29	867	42	32.7	3.1	0.2	39.4	1.7
Ko-5	5.8	8.8	0.19	15.56	0.43	34	40	26	5.5	276	21	17	1336	7.4	0.12	17	31	1194	31	24.9	8.3	0.4	3.3	1.7
Ko-6	5.3	8.8	0.25	20.13	0.07	36	39	26	5.5	260	18	17	1333	2.2	0.34	5	17	178	42	20.1	48.5	0.1	1.2	0.4
Ko-7	6.2	12.7	0.21	17.16	0.09	34	40	26	5.5	276	21	17	1336	2.6	0.23	5	41	166	514	20.2	16.3	0.1	1.5	0.5
Ko-8	5.8	10.7	0.41	30.21	0.09	35	36	29	5.5	307	40	18	1234	2.5	0.32	6.2	45	265	38	31.2	11.2	0.2	1.4	0.6
Ko-9	6.1	10.7	0.19	15.56	0.07	34	39	28	5.3	282	38	18	1265	1.8	1.8	5.8	41	385	36	19.5	3.9	0.3	0.7	0.3
Ko-10	6.1	5.7	0.30	23.49	0.09	35	38	27	5.6	284	34	17	1286	1.7	2.1	5.4	38	421	31	15.1	4.8	0.3	0.6	0.5
Ko-11	5.3	8	0.51	36.17	0.16	34	38	27	5.4	293	29	17	1279	2.4	2.1	46	54	271	23	26.2	38.4	0.3	2.1	0.4
Ko-12	5.4	11.7	0.49	34.97	0.17	33	40	26	5.4	282	29	18	1271	3.8	2.7	19	31	267	36	23.7	46.0	0.4	3.4	1.6
Ko-13	6.8	6	0.36	27.23	0.18	34	38	29	5.4	299	26	14	1296	2.8	2.9	29	43	245	32	28.4	124.0	0.8	9.4	0.8
Ko-14	6.4	5	0.16	12.96	0.08	35	39	25	5.7	233	19	13	1323	1.8	1.9	5	40	394	35	17.2	4.4	0.1	0.6	0.3
Ko-15	5.8	10.1	0.38	28.43	0.09	38	36	25	5.3	350	89	25	1189	1.9	3	5.2	11	39	10	12.4	219.0	0.3	2.1	0.6
Ko-16	6.0	10.4	0.26	20.83	0.1	38	36	25	5.3	350	89	25	1189	2.2	2.8	5.3	37	139	21	24.7	137.0	0.8	2.4	0.4
Ko-17	5.2	11.3	0.29	22.84	0.08	38	36	27	5.4	351	91	25	1216	2.5	0.9	21	12	54	10	16.8	179.0	0.2	2.9	0.7
Ko-18	6.0	3.6	0.25	20.13	0.07	37	38	24	5.2	366	81	21	1181	1.7	2.1	7	35	119	18	31.7	103.0	0.2	5.4	0.2
Ko-19	6.4	8.5	0.28	22.18	0.16	37	38	24	5.2	366	81	21	1181	2.9	4.6	21	22	117	15	28.1	485.0	0.6	3.2	0.4
Ko-20	5.5	10.5	0.27	21.51	0.1	35	40	25	5.2	351	92	19	1151	2	1.7	36	43	241	14	16.1	29.3	0.2	1.8	0.3
Ko-21	5.9	8.8	0.27	21.51	0.09	35	36	28	5.7	310	33	17	1287	2.5	1.7	44	36	136	15	16.2	26.5	0.2	5.7	0.5
Ko-22	5.6	9	0.27	21.51	0.05	35	38	27	5.5	331	31	17	1280	1.8	0.33	5	38	273	39	12.7	10.4	1.2	0.8	0.4

[a] ID represents C. indicum collection sites, where Ko refers to Kochi Prefecture.

[b] These parameter were measured during the field investigation.

[c] Soil mosture was calculated from SM150T output.

Table S2. Various conditioning factors for predicting habitat suitability map using MaxEnt model

	Topographic Factors									Soil Factors												Environmental Factors			Climatic Factors							
No	Slope	DEM	Aspect	Curvature	Plan Curvature	Profile Curvature	Hillshade	TWI	Elevation	pH	SM150T Output [a]	Soil Bearing Capacity [a]	EC	Soil Bulk Density	CEC	OCD	OCC	pH in H_2O	Clay	Sand	Silt	Dist. to Stream	Dist. to Urban	Dist. to Road	Annual Temperature	Annual Precipitation	BIO01	BIO05	BIO06	BIO12	BIO13	BIO14
0	18.76	190	137.39	0	0.160	0.160	112	5.57	55.39	6.16	0.304	9.98	0.19	1316	15	283	29	5.5	26	39	35	100.11	91.78	369.29	16.23	2035	16.23	30.0	1.5	2035	315	54
1	18.76	190	137.39	0	0.160	0.160	112	5.57	55.98	6.16	0.303	9.98	0.19	1316	15	283	29	5.5	26	39	35	100.11	91.78	369.29	16.23	2035	16.23	30.0	1.5	2035	315	54
2	18.76	190	137.39	0	0.160	0.160	112	5.57	63.74	6.16	0.304	9.98	0.19	1316	15	283	29	5.5	26	39	35	100.11	91.78	369.29	16.23	2035	16.23	30.0	1.5	2035	315	54
3	18.76	190	137.39	0	0.160	0.160	112	5.57	66.41	6.16	0.304	9.98	0.19	1316	15	283	29	5.5	26	39	35	100.11	91.78	369.29	16.23	2035	16.23	30.0	1.5	2035	315	54
4	17.30	146	185.53	0.64	0.748	0.108	138	4.96	73.17	6.11	0.304	10.07	0.16	1310	15	281	31	5.4	26	39	34	138.14	24.87	234.24	15.83	2057	15.83	29.6	1.2	2057	320	54
5	17.30	146	185.53	0.64	0.748	0.108	138	4.96	74.81	6.11	0.304	10.07	0.16	1310	15	281	31	5.4	26	39	34	138.14	24.87	234.24	15.83	2057	15.83	29.6	1.2	2057	320	54
6	17.30	146	185.53	0.64	0.748	0.108	138	4.96	84.51	6.11	0.304	10.07	0.16	1310	15	281	31	5.4	26	39	34	138.14	24.87	234.24	15.83	2057	15.83	29.6	1.2	2057	320	54
7	17.30	146	185.53	0.64	0.748	0.108	138	4.96	84.91	6.11	0.304	10.07	0.16	1310	15	281	31	5.4	26	39	34	138.14	24.87	234.24	15.83	2057	15.83	29.6	1.2	2057	320	54
8	17.30	146	185.53	0.64	0.748	0.108	138	4.96	90.33	6.11	0.304	10.07	0.16	1310	15	281	31	5.4	26	39	34	138.14	24.87	234.24	15.83	2057	15.83	29.6	1.2	2057	320	54
9	17.30	146	185.53	0.64	0.748	0.108	138	4.96	94.27	6.11	0.304	10.07	0.16	1310	15	281	31	5.4	26	39	34	138.14	24.87	234.24	15.83	2057	15.83	29.6	1.2	2057	320	54
10	17.30	146	185.53	0.64	0.748	0.108	138	4.96	94.56	6.11	0.304	10.07	0.16	1310	15	281	31	5.4	26	39	34	138.14	24.87	234.24	15.83	2057	15.83	29.6	1.2	2057	320	54
11	17.30	146	185.53	0.64	0.748	0.108	138	4.96	95.07	6.11	0.304	10.07	0.16	1310	15	281	31	5.4	26	39	34	138.14	24.87	234.24	15.83	2057	15.83	29.6	1.2	2057	320	54
12	17.30	146	185.53	0.64	0.748	0.108	138	4.96	95.58	6.11	0.304	10.07	0.16	1310	15	281	31	5.4	26	39	34	138.14	24.87	234.24	15.83	2057	15.83	29.6	1.2	2057	320	54
13	17.30	146	185.53	0.64	0.748	0.108	138	4.96	95.03	6.11	0.304	10.07	0.16	1310	15	281	31	5.4	26	39	34	138.14	24.87	234.24	15.83	2057	15.83	29.6	1.2	2057	320	54
14	17.30	146	185.53	0.64	0.748	0.108	138	4.96	97.25	6.11	0.304	10.07	0.16	1310	15	281	31	5.4	26	39	34	138.14	24.87	234.24	15.83	2057	15.83	29.6	1.2	2057	320	54
15	17.30	146	185.53	0.64	0.748	0.108	138	4.96	99.13	6.11	0.304	10.07	0.16	1310	15	281	31	5.4	26	39	34	138.14	24.87	234.24	15.83	2057	15.83	29.6	1.2	2057	320	54
16	17.30	146	185.53	0.64	0.748	0.108	138	4.96	105.33	6.11	0.304	10.07	0.16	1310	15	281	31	5.4	26	39	34	138.14	24.87	234.24	15.83	2057	15.83	29.6	1.2	2057	320	54
17	17.30	146	185.53	0.64	0.748	0.108	138	4.96	109.14	6.11	0.304	10.07	0.16	1310	15	281	31	5.4	26	39	34	138.14	24.87	234.24	15.83	2057	15.83	29.6	1.2	2057	320	54
18	34.98	156	239.04	-1.28	-0.358	0.922	172	4.22	107.96	6.05	0.309	9.96	0.16	1301	16	302	31	5.4	27	39	35	293.45	15.86	55.23	15.83	2057	15.83	29.6	1.2	2057	320	54
19	34.98	156	239.04	-1.28	-0.358	0.922	172	4.22	107.94	6.05	0.309	9.96	0.16	1301	16	302	31	5.4	27	39	35	293.45	15.86	55.23	15.83	2057	15.83	29.6	1.2	2057	320	54
20	34.98	156	239.04	-1.28	-0.358	0.922	172	4.22	107.42	6.05	0.309	9.96	0.16	1301	16	302	31	5.4	27	39	35	293.45	15.86	55.23	15.83	2057	15.83	29.6	1.2	2057	320	54
21	34.98	156	239.04	-1.28	-0.358	0.922	172	4.22	109.84	6.05	0.309	9.96	0.16	1301	16	302	31	5.5	27	39	35	293.45	15.86	55.23	15.83	2057	15.83	29.6	1.2	2057	320	54
22	34.98	156	239.04	-1.28	-0.358	0.922	172	4.22	113.44	6.05	0.309	9.96	0.16	1301	16	302	31	5.5	27	39	35	293.45	15.86	55.23	15.83	2057	15.83	29.6	1.2	2057	320	54
23	34.98	156	239.04	-1.28	-0.358	0.922	172	4.22	114.35	6.05	0.309	9.96	0.16	1301	16	302	31	5.5	27	39	35	293.45	15.86	55.23	15.83	2057	15.83	29.6	1.2	2057	320	54
24	34.98	156	239.04	-1.28	-0.358	0.922	172	4.22	115.48	6.05	0.309	9.96	0.16	1301	16	302	31	5.5	27	39	35	293.45	15.86	55.23	15.83	2057	15.83	29.6	1.2	2057	320	54
25	34.98	156	239.04	-1.28	-0.358	0.922	172	4.22	114.86	6.05	0.309	9.96	0.16	1301	16	302	31	5.5	27	39	35	293.45	15.86	55.23	15.83	2057	15.83	29.6	1.2	2057	320	54
26	34.98	156	239.04	-1.28	-0.358	0.922	172	4.22	113.1	6.05	0.309	9.96	0.16	1301	16	302	31	5.5	27	39	35	293.45	15.86	55.23	15.83	2057	15.83	29.6	1.2	2057	320	54
27	18.99	125	234.46	-1.28	-0.640	0.640	180	6.25	91.75	6.25	0.238	10.39	0.18	1343	13	280	22	5.5	25	41	34	483.53	17.34	476.04	16.23	2035	16.23	30.0	1.5	2035	315	54
28	18.99	125	234.46	-1.28	-0.640	0.640	180	6.25	91.54	6.25	0.238	10.39	0.18	1343	13	280	22	5.5	25	41	34	483.53	17.34	476.04	16.23	2035	16.23	30.0	1.5	2035	315	54
29	7.43	153	122.47	0.64	0.345	-0.295	156	6.73	92.57	6.18	0.260	10.47	0.18	1338	14	289	23	5.4	25	41	34	475.77	16.89	472.60	16.23	2035	16.23	30.0	1.5	2035	315	54
30	20.37	169	152.74	0	-0.384	-0.384	109	5.71	93.99	6.16	0.263	10.42	0.18	1323	13	295	25	5.5	25	39	35	465.62	19.09	479.41	16.23	2035	16.23	30.0	1.5	2035	315	54
31	20.37	169	152.74	0	-0.384	-0.384	109	5.71	92.13	6.16	0.263	10.42	0.18	1323	13	295	25	5.5	25	39	35	465.62	19.09	479.41	16.23	2035	16.23	30.0	1.5	2035	315	54
32	20.37	169	152.74	0	-0.384	-0.384	109	5.71	92.07	6.16	0.263	10.42	0.18	1323	13	295	25	5.5	25	39	35	465.62	19.09	479.41	16.23	2035	16.23	30.0	1.5	2035	315	54
33	20.37	169	152.74	0	-0.384	-0.384	109	5.71	92.85	6.16	0.263	10.42	0.18	1323	13	295	25	5.5	25	39	35	465.62	19.09	479.41	16.23	2035	16.23	30.0	1.5	2035	315	54
34	5.71	92	0.00	1.92	1.280	-0.640	192	5.38	64.6	6.15	0.227	10.65	0.19	1302	16	265	28	5.6	27	39	35	414.19	219.86	161.17	16.95	2025	16.95	31.0	1.8	2025	313	53
35	5.71	92	0.00	1.92	1.280	-0.640	192	5.38	64.48	6.15	0.227	10.65	0.19	1302	16	265	28	5.6	27	39	35	414.19	219.86	161.17	16.95	2025	16.95	31.0	1.8	2025	313	53
36	5.71	92	0.00	1.92	1.280	-0.640	192	5.38	64.33	6.15	0.227	10.65	0.19	1302	16	265	28	5.6	27	39	35	414.19	219.86	161.17	16.95	2025	16.95	31.0	1.8	2025	313	53
37	5.71	92	0.00	1.92	1.280	-0.640	192	5.38	64.53	6.15	0.227	10.65	0.19	1302	16	265	28	5.6	27	39	35	414.19	219.86	161.17	16.95	2025	16.95	31.0	1.8	2025	313	53
38	5.71	92	0.00	1.92	1.280	-0.640	192	5.38	65.93	6.15	0.227	10.65	0.19	1302	16	265	28	5.6	27	39	35	414.19	219.86	161.17	16.95	2025	16.95	31.0	1.8	2025	313	53
39	5.71	92	0.00	1.92	1.280	-0.640	192	5.38	67.36	6.15	0.227	10.65	0.19	1302	16	265	28	5.6	27	39	35	414.19	219.86	161.17	16.95	2025	16.95	31.0	1.8	2025	313	53
40	27.83	263	307.30	0.64	0.221	-0.419	242	4.87	204.04	6.25	0.260	10.99	0.14	1317	15	265	25	5.4	25	40	35	939.40	667.76	696.90	16.53	2055	16.53	30.6	1.4	2055	321	54
41	14.62	225	237.53	0	0.000	0.000	184	7.53	200.16	6.33	0.290	11.19	0.14	1338	15	248	25	5.5	26	40	35	1023.64	656.59	794.71	16.53	2055	16.53	30.6	1.4	2055	321	54
42	14.62	225	237.53	0	0.000	0.000	184	7.53	193.87	6.33	0.290	11.19	0.14	1338	15	248	25	5.5	26	40	35	1023.64	656.59	794.71	16.53	2055	16.53	30.6	1.4	2055	321	54
43	24.29	154	12.80	-1.28	-0.640	0.640	203	5.30	214.1	6.02	0.193	10.14	0.23	1336	17	276	21	5.5	26	40	34	1003.32	697.82	629.27	16.53	2055	16.53	30.6	1.4	2055	321	54
44	32.00	172	309.81	0	-0.315	-0.315	248	5.70	207.17	6.05	0.234	10.48	0.18	1339	17	264	20	5.6	25	41	34	886.42	663.67	788.53	16.53	2055	16.53	30.6	1.4	2055	321	54
45	32.00	172	309.81	0	-0.315	-0.315	248	5.70	201.94	6.05	0.234	10.48	0.18	1339	17	264	20	5.6	25	41	34	886.42	663.67	788.53	16.53	2055	16.53	30.6	1.4	2055	321	54
46	30.96	335	323.13	-0.64	-0.410	0.230	246	5.45	318.18	6.71	0.098	16.60	0.15	1287	16	289	28	5.4	28	37	35	1790.89	97.71	239.41	15.94	2059	15.94	30.0	0.9	2059	321	54
47*	30.96	335	323.13	-0.64	-0.410	0.230	246	5.45	326.07	6.71	0.110	16.80	0.15	1287	16	289	28	5.4	28	37	35	1790.89	97.71	239.41	15.94	2059	15.94	30.0	0.9	2059	321	54
48*	24.54	269	331.19	-0.64	-0.512	0.128	235	7.00	296.08	7.59	0.245	9.78	0.43	1304	17	293	26	5.6	25	40	35	1327.87	223.79	406.32	16.28	2054	16.28	30.3	1.2	2054	320	54
49*	14.62	225	237.53	0	0.000	0.000	184	7.53	196.97	6.33	0.139	10.29	0.14	1338	15	248	25	5.5	26	40	35	1023.64	656.59	794.71	16.53	2055	16.53	30.6	1.4	2055	321	54
50*	14.62	225	237.53	0	0.000	0.000	184	7.53	198.86	6.33	0.390	11.99	0.14	1338	15	248	25	5.5	26	40	35	1023.64	656.59	794.71	16.53	2055	16.53	30.6	1.4	2055	321	54
51*	24.29	154	12.80	-1.28	-0.640	0.640	203	5.30	225.67	6.02	0.189	8.78	0.23	1336	17	276	21	5.5	26	40	34	1003.32	697.82	629.27	16.53	2055	16.53	30.6	1.4	2055	321	54
52*	23.84	219	354.81	0	-0.630	-0.630	220	6.64	220.11	5.61	0.247	8.77	0.16	1333	17	260	18	5.5	26	39	36	964.59	715.00	662.72	16.53	2055	16.53	30.6	1.4	2055	321	54
53*	24.29	154	12.80	-1.28	-0.640	0.640	203	5.30	231.71	6.02	0.209	12.70	0.23	1336	17	276	21	5.5	26	40	34	1003.32	697.82	629.27	16.53	2055	16.53	30.6	1.4	2055	321	54
54	23.84	219	354.81	0	-0.630	-0.630	220	6.64	227.81	5.61	0.213	9.62	0.16	1333	17	260	18	5.5	26	39	36	964.59	715.00	662.72	16.53	2055	16.53	30.6	1.4	2055	321	54
55	23.84	219	354.81	0	-0.630	-0.630	220	6.64	227.33	5.61	0.213	9.62	0.16	1333	17	260	18	5.5	26	39	36	964.59	715.00	662.72	16.53	2055	16.53	30.6	1.4	2055	321	54
56	24.29	154	12.80	-1.28	-0.640	0.640	203	5.30	229.51	6.02	0.193	10.14	0.23	1336	17	276	21	5.5	26	40	34	1003.32	697.82	629.27	16.53	2055	16.53	30.6	1.4	2055	321	54
57	24.29	154	12.80	-1.28	-0.640	0.640	203	5.30	229.47	6.02	0.193	10.14	0.23	1336	17	276	21	5.5	26	40	34	1003.32	697.82	629.27	16.53	2055	16.53	30.6	1.4	2055	321	54
58	24.29	154	12.80	-1.28	-0.640	0.640	203	5.30	229.48	6.02	0.193	10.14	0.23	1336	17	276	21	5.5	26	40	34	1003.32	697.82	629.27	16.53	2055	16.53	30.6	1.4	2055	321	54
59	24.29	154	12.80	-1.28	-0.640	0.640	203	5.30	226.55	6.02	0.193	10.14	0.23	1336	17	276	21	5.5	26	40	34	1003.32	697.82	629.27	16.53	2055	16.53	30.6	1.4	2055	321	54
60	24.29	154	12.80	-1.28	-0.640	0.640	203	5.30	223.41	6.02	0.193	10.14	0.23	1336	17	276	21	5.5	26	40	34	1003.32	697.82	629.27	16.53	2055	16.53	30.6	1.4	2055	321	54
61	23.84	219	354.81	0	-0.630	-0.630	220	6.64	219.91	5.61	0.213	9.62	0.16	1333	17	260	18	5.5	26	39	36	964.59	715.00	662.72	16.53	2055	16.53	30.6	1.4	2055	321	54
62	14.62	225	237.53	0	0.000	0.000	184	7.53	203.39	6.33	0.209	11.19	0.14	1338	15	248	25	5.5	26	40	35	1023.64	656.59	794.71	16.53	2055	16.53	30.6	1.4	2055	321	54
63	14.62	225	237.53	0	0.000	0.000	184	7.53	197.58	6.33	0.290	11.19	0.14	1338	15	248	25	5.5	26	40	35	1023.64	656.59	794.71	16.53	2055	16.53	30.6	1.4	2055	321	54
64	14.62	225	237.53	0	0.000	0.000	184	7.53	196.65	6.33	0.290	11.19	0.14	1338	15	248	25	5.5	26	40	35	1023.64	656.59	794.71	16.53	2055	16.53	30.6	1.4	2055	321	54
65*	15.68	209	175.91	-1.28	-0.550	0.730	136	6.44	140.59	5.86	0.410	10.68	0.10	1234	18	307	40	5.5	29	36	35	72.56	138.39	399.80	15.71	2017	15.71	29.7	0.9	2017	315	52

66	15.68	209	175.91	-1.28	-0.550	0.730	136	6.44	135.42	5.86	0.441	10.08	0.10	1234	18	307	40	5.5	29	36	35	72.56	138.39	399.80	15.71	2017	15.71	29.7	0.9	2017	315	52
67	23.71	244	120.07	-1.28	-0.640	0.640	95	7.20	183.43	6.03	0.256	8.95	0.09	1275	17	277	29	5.3	28	38	34	406.70	372.63	745.28	15.25	2042	15.25	29.3	0.5	2042	320	52
68*	37.58	315	117.90	1.28	0.905	-0.375	37	4.55	190.92	6.04	0.185	10.70	0.08	1265	18	282	38	5.3	28	39	34	456.72	410.85	801.95	15.25	2042	15.25	29.3	0.5	2042	320	52
69	23.77	234	219.47	-1.28	-0.640	0.640	158	5.04	215.15	6.26	0.309	6.10	0.09	1286	17	284	34	5.6	27	38	35	400.38	379.54	727.68	15.18	2045	15.18	29.2	0.4	2045	322	52
70*	23.77	234	219.47	-1.28	-0.640	0.640	158	5.04	213.96	6.26	0.300	5.66	0.09	1286	17	284	34	5.6	27	38	35	400.38	379.54	727.68	15.18	2045	15.18	29.2	0.4	2045	322	52
71*	41.18	388	100.54	0	-0.113	-0.113	37	5.29	301.08	5.40	0.510	8.01	0.16	1279	17	293	29	5.4	27	38	34	366.34	613.40	985.11	15.18	2045	15.18	29.2	0.4	2045	322	52
72	41.18	388	100.54	0	-0.113	-0.113	37	5.29	301.34	5.40	0.616	8.10	0.16	1279	17	293	29	5.4	27	38	34	366.34	613.40	985.11	15.18	2045	15.18	29.2	0.4	2045	322	52
73	31.33	372	227.66	0.64	0.449	-0.191	158	5.26	320.34	5.47	0.479	10.14	0.14	1267	17	285	31	5.4	26	39	34	446.54	541.30	876.91	15.18	2045	15.18	29.2	0.4	2045	322	52
74*	18.58	451	202.75	-0.64	-0.666	-0.026	149	7.28	321.51	5.25	0.485	11.66	0.16	1271	18	282	29	5.4	26	40	33	480.34	593.92	934.06	15.18	2045	15.18	29.2	0.4	2045	322	52
75*	30.02	142	66.54	-1.28	0.149	1.429	123	4.79	86.26	5.28	0.359	6.00	0.17	1296	14	299	26	5.4	29	38	34	98.03	92.08	431.26	16.07	1995	16.07	30.0	1.2	1995	311	52
76	6.84	172	180.00	-0.64	0.000	0.640	163	6.30	87.26	5.36	0.386	6.67	0.16	1269	18	307	35	5.6	29	36	34	99.53	93.32	431.83	16.07	1995	16.07	30.0	1.2	1995	311	52
77	6.84	172	180.00	-0.64	0.000	0.640	163	6.30	87.26	5.36	0.386	6.67	0.16	1269	18	307	35	5.6	29	36	34	99.53	93.32	431.83	16.07	1995	16.07	30.0	1.2	1995	311	52
78*	7.43	96	94.40	0.64	0.000	-0.640	161	5.81	175.51	6.10	0.157	5.02	0.08	1323	13	233	19	5.7	25	39	35	137.19	96.14	81.51	16.63	2000	16.63	30.6	1.7	2000	311	53
79	22.38	369	330.95	0	0.338	0.338	232	4.00	213.82	4.64	0.326	10.37	0.09	1219	20	340	34	5.2	28	37	36	121.92	56.30	192.60	14.26	2049	14.26	28.2	-0.3	2049	320	52
80	30.77	343	49.09	3.2	1.729	-1.471	148	5.97	235.24	4.62	0.335	10.58	0.09	1213	24	342	48	5.2	28	37	36	103.10	85.49	241.03	14.26	2049	14.26	28.2	-0.3	2049	320	52
81*	24.73	402	117.12	1.28	0.518	-0.762	91	5.51	275.06	4.73	0.379	10.07	0.09	1189	25	350	89	5.3	25	36	38	127.34	85.56	237.08	14.26	2049	14.26	28.2	-0.3	2049	320	52
82	24.73	402	117.12	1.28	0.518	-0.762	91	5.51	270.06	4.73	0.309	10.37	0.09	1189	25	350	89	5.3	25	36	38	127.34	85.56	237.08	14.26	2049	14.26	28.2	-0.3	2049	320	52
83	24.73	402	117.12	1.28	0.518	-0.762	91	5.51	278.03	4.73	0.309	10.37	0.09	1189	25	350	89	5.3	25	36	38	127.34	85.56	237.08	14.26	2049	14.26	28.2	-0.3	2049	320	52
84	24.73	402	117.12	1.28	0.518	-0.762	91	5.51	264.93	4.73	0.309	10.37	0.09	1189	25	350	89	5.3	25	36	38	127.34	85.56	237.08	14.26	2049	14.26	28.2	-0.3	2049	320	52
85*	24.73	402	117.12	1.28	0.518	-0.762	91	5.51	262.65	4.73	0.259	10.37	0.09	1189	25	350	89	5.3	25	36	38	127.34	85.56	237.08	14.26	2049	14.26	28.2	-0.3	2049	320	52
86	24.73	402	117.12	1.28	0.518	-0.762	91	5.51	268.07	4.73	0.309	10.37	0.09	1189	25	350	89	5.3	25	36	38	127.34	85.56	237.08	14.26	2049	14.26	28.2	-0.3	2049	320	52
87*	24.73	402	117.12	1.28	0.518	-0.762	91	5.51	272.54	4.73	0.289	11.29	0.09	1189	25	350	89	5.3	25	36	38	127.34	85.56	237.08	14.26	2049	14.26	28.2	-0.3	2049	320	52
88	24.73	402	117.12	1.28	0.518	-0.762	91	5.51	272.98	4.73	0.309	10.37	0.09	1189	25	350	89	5.3	25	36	38	127.34	85.56	237.08	14.26	2049	14.26	28.2	-0.3	2049	320	52
89	34.94	445	23.63	-0.64	-0.302	0.338	185	5.48	273.37	4.61	0.324	10.80	0.09	1216	25	351	91	5.4	27	36	38	231.34	39.83	82.53	14.26	2049	14.26	28.2	-0.3	2049	320	52
90	34.94	445	23.63	-0.64	-0.302	0.338	185	5.48	277.99	4.61	0.324	10.80	0.09	1216	25	351	91	5.4	27	36	38	231.34	39.83	82.53	14.26	2049	14.26	28.2	-0.3	2049	320	52
91	2.92	289	101.31	-1.92	-1.280	0.640	172	11.51	294.35	4.71	0.258	6.01	0.11	1181	21	366	81	5.2	24	38	37	181.43	173.42	408.47	13.93	2073	13.93	27.9	-0.6	2073	325	52
92*	2.92	289	101.31	-1.92	-1.280	0.640	172	11.51	291.5	4.71	0.248	3.55	0.11	1181	21	366	81	5.2	24	38	37	181.43	173.42	408.47	13.93	2073	13.93	27.9	-0.6	2073	325	52
93*	2.92	289	101.31	-1.92	-1.280	0.640	172	11.51	292.89	4.71	0.278	8.46	0.11	1181	21	366	81	5.2	24	38	37	181.43	173.42	408.47	13.93	2073	13.93	27.9	-0.6	2073	325	52
94*	4.36	325	113.20	0.64	0.640	0.000	167	5.65	296.02	5.29	0.268	10.45	0.10	1151	19	351	92	5.2	25	40	35	74.36	303.58	196.52	14.00	2078	14.00	27.9	-0.5	2078	326	53
95*	5.11	347	153.43	0.64	0.512	-0.128	164	5.49	97.12	5.11	0.266	8.80	0.09	1287	17	310	33	5.7	28	36	35	65.95	191.58	570.44	15.18	2045	15.18	29.2	0.4	2045	322	52
96	5.11	347	153.43	0.64	0.512	-0.128	164	5.49	92.24	5.11	0.263	8.80	0.09	1287	17	310	33	5.7	28	36	35	65.95	191.58	570.44	15.18	2045	15.18	29.2	0.4	2045	322	52
97	15.68	209	175.91	-1.28	-0.550	0.730	136	6.44	97.15	5.86	0.441	10.08	0.10	1234	18	307	40	5.5	29	36	35	72.56	138.39	399.80	15.71	2017	15.71	29.7	0.9	2017	315	52
98	21.53	228	59.53	-1.28	-0.368	0.912	151	7.44	70.79	5.65	0.276	8.85	0.07	1293	14	296	22	5.7	26	39	34	231.49	301.45	593.39	16.33	1981	16.33	30.3	1.5	1981	309	52
99	14.20	129	161.57	0	-0.192	-0.192	135	7.60	68.74	5.65	0.285	8.69	0.09	1318	15	254	22	5.6	26	39	35	236.28	306.20	579.28	16.33	1981	16.33	30.3	1.5	1981	309	52
100	27.55	291	257.83	-1.92	-1.217	0.703	205	4.88	129.28	5.21	0.217	9.19	0.11	1270	22	293	44	5.3	27	40	34	168.83	99.38	1519.54	15.55	2022	15.55	29.6	0.7	2022	318	53
101	9.09	223	180.00	-1.28	-1.280	0.000	157	14.46	117.23	5.38	0.241	9.08	0.10	1293	18	295	32	5.2	27	40	34	114.99	168.15	1390.94	15.55	2022	15.55	29.6	0.7	2022	318	53
102*	16.82	182	235.78	0	-0.148	-0.148	182	6.37	75.31	5.69	0.265	8.99	0.05	1280	17	331	31	5.5	27	38	35	59.28	127.62	918.56	15.55	2022	15.55	29.6	0.7	2022	318	53
103	7.60	153	192.99	1.28	0.544	-0.736	166	5.10	130.04	5.46	0.368	8.89	0.11	1236	18	544	33	5.3	27	36	36	57.86	62.20	115.15	15.68	2000	15.68	29.6	1	2000	313	53
104	18.03	193	227.49	0	0.000	0.000	173	6.79	140.55	5.19	0.351	9.17	0.11	1258	18	321	32	5.1	26	39	36	30.51	77.21	80.51	15.08	2044	15.08	28.9	0.5	2044	321	53
105	24.06	345	124.05	0.64	0.049	-0.591	92	6.63	167.24	5.40	0.360	9.28	0.12	1273	18	326	29	5.4	27	38	34	88.76	90.77	553.00	15.19	2057	15.19	29.0	0.6	2057	321	54
106	24.06	345	124.05	0.64	0.049	-0.591	92	6.63	168.26	5.40	0.360	9.28	0.12	1273	18	326	29	5.4	27	38	34	88.76	90.77	553.00	15.19	2057	15.19	29.0	0.6	2057	321	54
107	26.64	278	113.50	-1.28	-0.881	0.399	85	10.81	175.36	5.81	0.335	9.26	0.14	1293	15	319	28	5.3	26	40	34	79.69	120.98	563.58	15.19	2057	15.19	29.0	0.6	2057	321	54
108	11.79	347	196.70	0.64	-0.132	-0.772	159	5.35	219.62	5.88	0.322	9.42	0.14	1265	21	359	32	5.1	28	38	35	360.16	67.02	305.75	15.02	2093	15.02	28.8	0.4	2093	327	54
109	32.06	399	250.41	-1.28	-1.120	0.160	193	6.67	267.72	5.96	0.331	9.64	0.15	1287	20	327	28	5.4	28	38	33	1023.14	116.32	449.93	15.18	2078	15.18	28.9	0.6	2078	325	54
110	12.60	282	206.57	1.28	0.640	-0.640	163	5.28	132.34	5.99	0.325	9.73	0.15	1293	16	325	32	5.5	27	38	35	929.18	139.75	20.71	15.18	2078	15.18	28.9	0.6	2078	325	54
111	18.87	231	249.44	-0.64	-0.982	-0.342	194	9.18	171.11	5.91	0.209	10.26	0.19	1340	18	270	21	5.6	27	41	33	932.59	638.40	582.60	16.81	2067	16.81	30.8	1.6	2067	321	54
112	24.29	154	12.80	-1.28	-0.640	0.640	203	5.30	211.67	6.02	0.193	10.14	0.23	1336	17	276	21	5.5	26	40	34	1003.32	697.82	629.27	16.53	2055	16.53	30.6	1.4	2055	321	54
113	14.62	225	237.53	0	0.000	0.000	184	7.53	184.22	6.33	0.290	11.19	0.14	1338	15	248	25	5.5	26	40	35	1023.64	656.59	794.71	16.53	2055	16.53	30.6	1.4	2055	321	54
114	30.96	335	323.13	-0.64	-0.410	0.230	246	5.45	313.91	6.71	0.098	16.60	0.15	1287	16	289	28	5.4	28	37	35	1790.89	97.71	239.41	15.94	2059	15.94	30.0	0.9	2059	321	54

Topographic Factors: *Slope, Digital Elevation Model (DEM), Aspect, Curvature, Plan curvature, Profile curvature, Elevation, TWI, Hillshade;* ***Soil Factors:*** *pH, SM150T Output, Soil Bearing Capacity, Electrical Conductivity (EC), Soil Bulk Density, Soil Cation Exchange Capacity (CEC), Soil Organic Carbon Density (OCD), Soil Organic Carbon Content (OCC), Soil pH in H2O, Soil Clay Content, Soil Sand Content, Soil Silt Content;* ***Environmental Factors:*** *Distance to Stream, Distance to Urban, Distance to Road;* ***Climatic Factors:*** *Annual Mean Precipitation, Annual Mean Temperature, Annual Mean Temperature (BIO1), Maximum Temperature of the Warmest Month (BIO5), Minimum Temperature of the Coldest Month (BIO6), Annual Precipitation (BIO12), Precipitation of the Wettest Month (BIO13), Precipitation of the Driest Month (BIO14)*

a These value were interporated using measured data marked by * in first column

Table S3. Conditioning factors used for prediction of habitat suitability map of *C. indicum*

Category	Conditioning Factors	Data Scale
Topographical factors	Slope (°)	Continuous
	Digital elevation model (DEM) (m)	Continuous
	Aspect (°)	Categorical (5 classes)
	Curvature (m^{-1})	Continuous
	Plan curvature (m^{-1})	Continuous
	Profile curvature (m^{-1})	Continuous
	Elevation (m)	Continuous
	Topographic Wetness Index (TWI)	Continuous
	Hillshade (°)	Continuous
Soil factors	pH	Continuous
	pH in H_2O	Continuous
	Electrical Conductivity (mS cm^{-1})	Continuous
	Soil Bearing Capacity (t sf^{-1})	Continuous
	Soil Moisture Sensor Output (V)	Continuous
	Soil Bulk Density (kg m^{-3})	Continuous
	Cation Exchange Capacity (CEC) (cmolc kg^{-1})	Continuous
	Clay Content (%)	Continuous
	Sand Content (%)	Continuous
	Silt Content (%)	Continuous
	Organic Carbon Density (OCD) (kg m^{-3})	Continuous
	Organic Carbon Content (OCC) (g kg^{-1})	Continuous
Climatic factors	Annual Mean Precipitation (mm)	Continuous
	Annual Mean Temperature (°C)	Continuous
Environmental factors	Distance to Stream (m)	Continuous
	Distance to Urban (m)	Continuous
	Distance to Road (m)	Continuous
Bioclimatic variables	Annual Mean Temperature (BIO1) (°C)	Continuous
	Maximum Temperature of the Warmest Month (BIO5) (°C)	Continuous
	Minimum Temperature of the Coldest Month (BIO6) (°C)	Continuous
	Annual Precipitation (BIO12) (mm $year^{-1}$)	Continuous
	Precipitation of the Wettest Month (BIO13) (mm $month^{-1}$)	Continuous
	Precipitation of the Driest Month (BIO14) (mm $month^{-1}$)	Continuous

Figure S1. Analysis of the relative importance of effective environmental variables

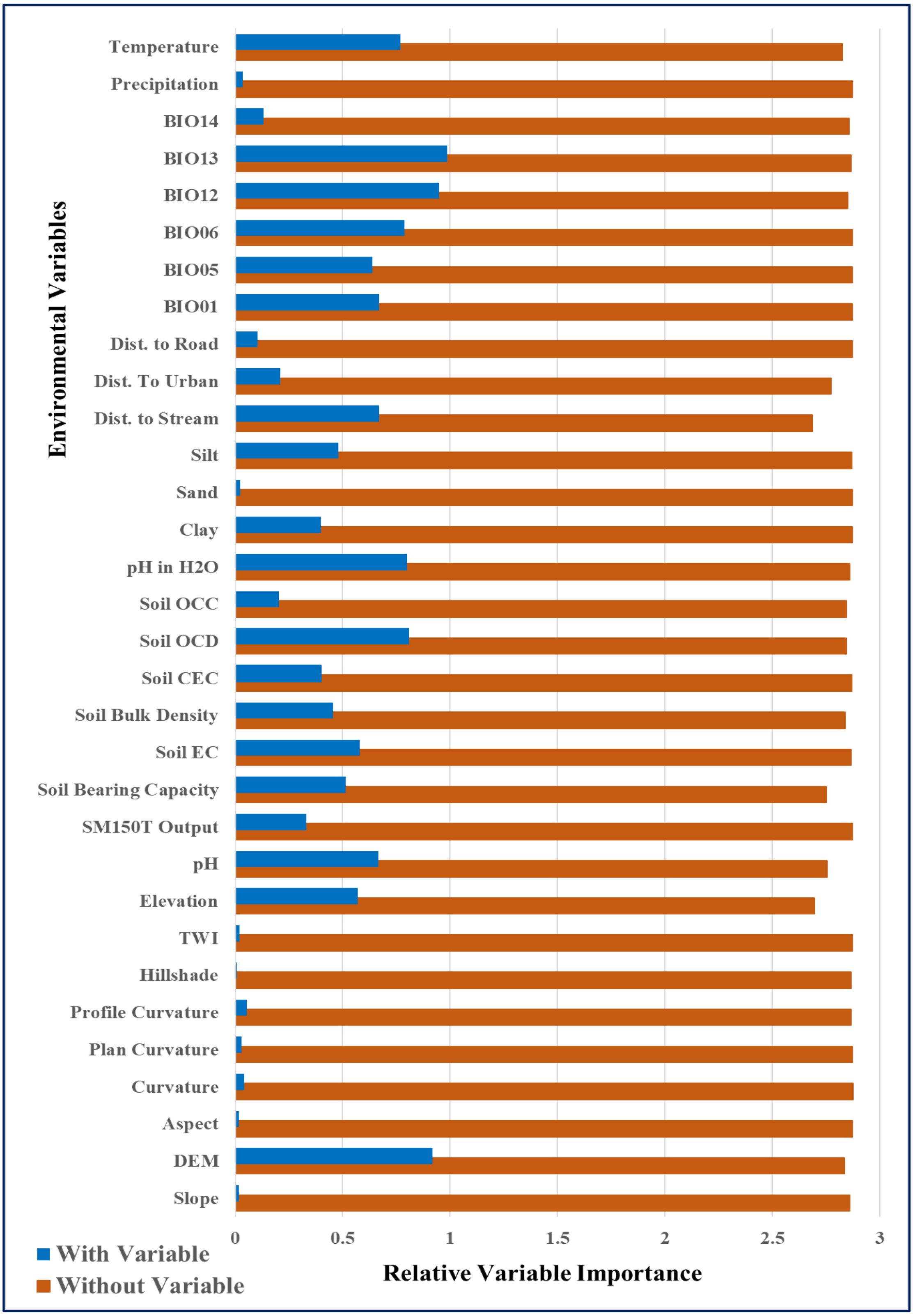

Topographical factors: *Slope, Digital Elevation Model (DEM), Aspect, Curvature, Plan Curvature, Profile Curvature, Elevation, Topographic Wetness Index (TWI), Hillshade;* ***Soil factors:*** *pH, pH in H_2O, Electrical Conductivity (EC), Soil Bearing Capacity, Soil Moisture Sensor Output (V)(SM150T Output), Soil Bulk Density, Cation Exchange Capacity (CEC), Clay Content, Sand Content, Silt Content, Organic Carbon Density (OCD), Organic Carbon Content (OCC);* ***Environmental factors;*** *Distance to Stream, Distance to Urban, Distance to Road;* ***Climatic factors;*** *Rainfall, Minimum Temperature, Maximum Temperature, Annual Mean Temperature (BIO01), Maximum Temperature of Warmest Month (BIO05), Minimum Temperature of Coldest Month (BIO06), Annual Precipitation (BIO12), Precipitation of Wettest Month (BIO13), Precipitation of Driest Month (BIO14)*

Figure S2. Photographs of *Chrysanthemum indium* L. in situ (at the collection sites)

Plant Location ID[a]	Longitude (E)	Latitude (N)	Elevation (m)
Ko-1	133° 43' 50.4"	33° 35' 33.12"	326
Ko-2	133° 43' 35.5"	33° 35' 20.82"	296
Ko-3	133° 43' 14.59"	33° 34' 48.65"	197
Ko-4	133° 43' 14.34"	33° 34' 48.1"	199
Ko-5	133° 42' 58.3"	33° 34' 38.06"	226
Ko-6	133° 42' 59.08"	33° 34' 35.9"	220
Ko-7	133° 42' 57.57"	33° 34' 38.28"	232
Ko-8	133° 44' 6.93"	33° 38' 28.55"	141
Ko-9	133° 43' 55.26"	33° 38' 36.82"	191
Ko-10	133° 43' 59.79"	33° 38' 33.49"	214
Ko-11	133° 44' 21.54"	33° 38' 46.85"	301
Ko-12	133° 44' 11.05"	33° 38' 44.76"	322
Ko-13	133° 43' 49.03"	33° 38' 19.7"	86
Ko-14	133° 43' 6.83"	33° 37' 18.78"	176
Ko-15	133° 52' 20.23"	33° 40' 50.78"	275
Ko-16	133° 52' 23.51"	33° 40' 50.72"	263
Ko-17	133° 52' 18.18"	33° 40' 50"	273
Ko-18	133° 52' 36.35"	33° 40' 30.73"	292
Ko-19	133° 52' 36.33"	33° 40' 30.54"	293
Ko-20	133° 52' 59.64"	33° 40' 5.54"	296
Ko-21	133° 44' 24.09"	33° 38' 32.58"	97
Ko-22	133° 43' 8.01"	33° 38' 35.53"	75

[a] ID represents the location of *C. indicum* collection in which Ko refers to Kochi prefecture.

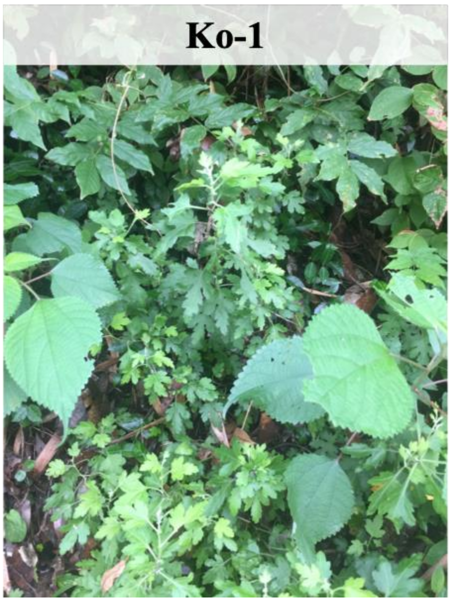

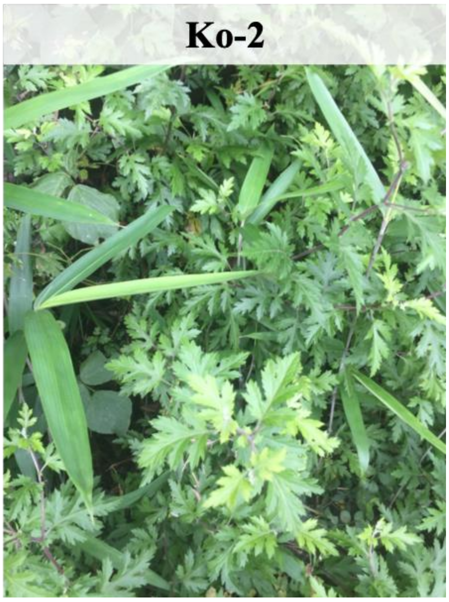

Ko-3

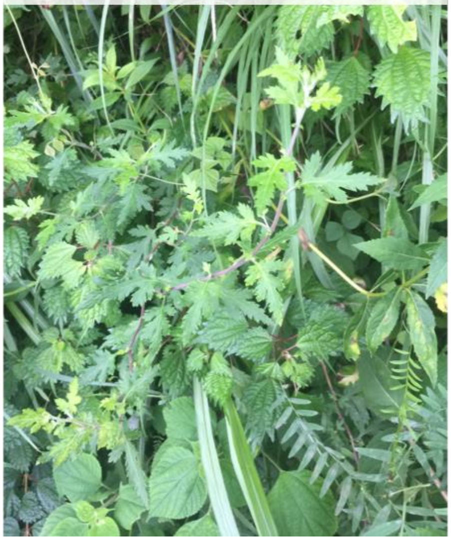

Ko-4

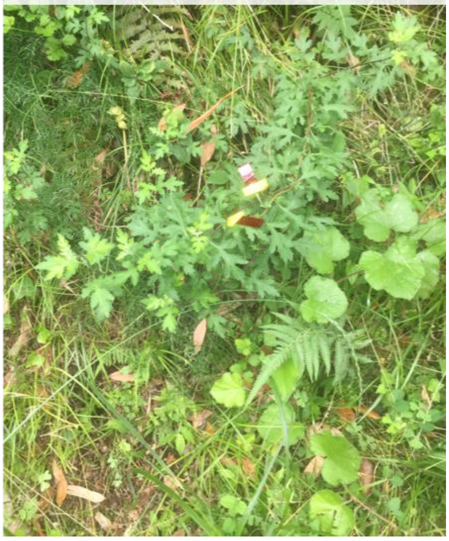

Ko-5

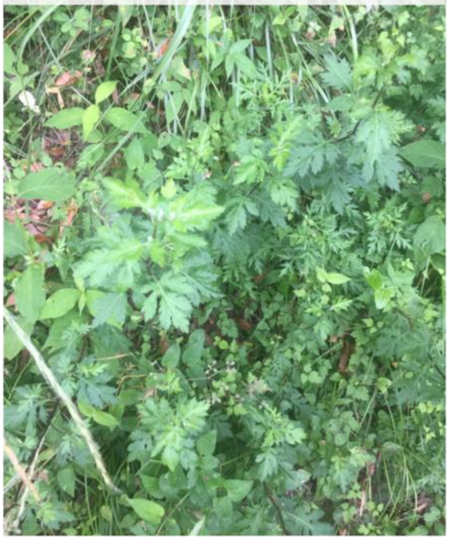

Ko-6

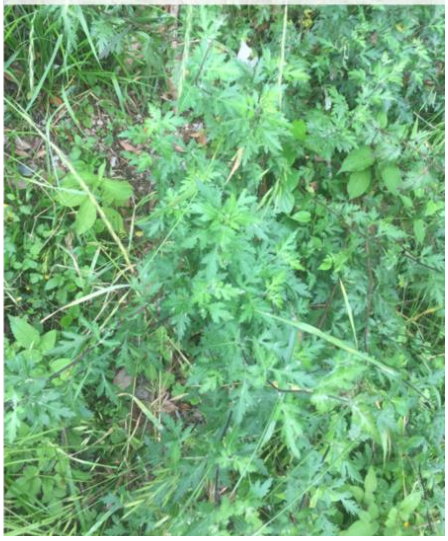

Ko-7

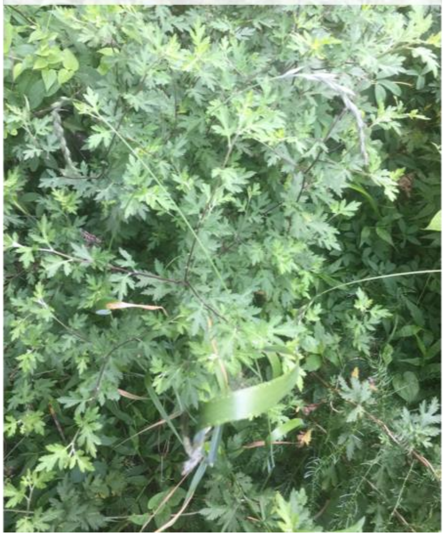

Ko-8

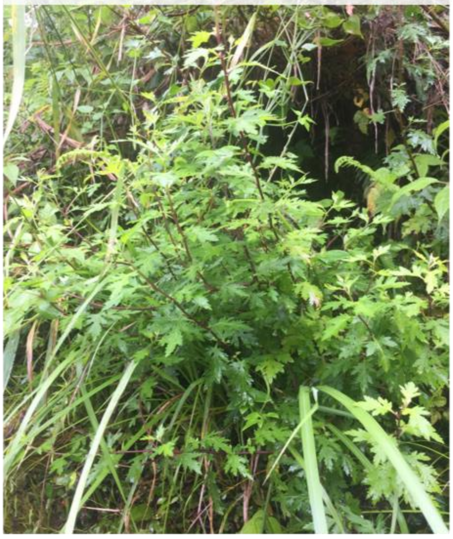

Ko-9

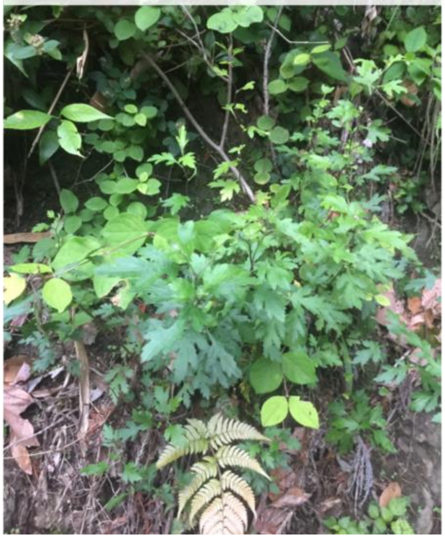

Ko-10

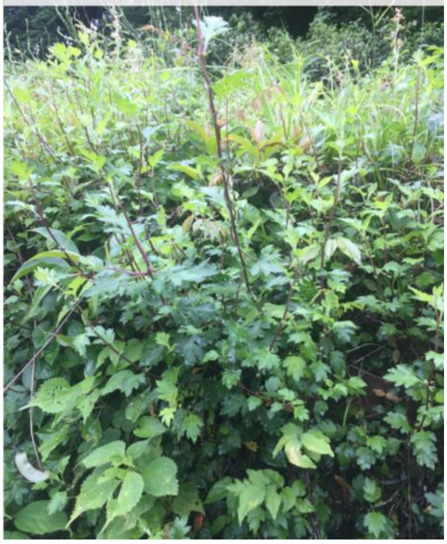

Ko-11

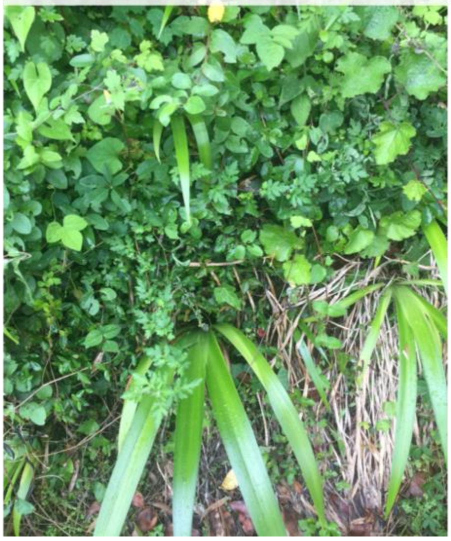

Ko-12

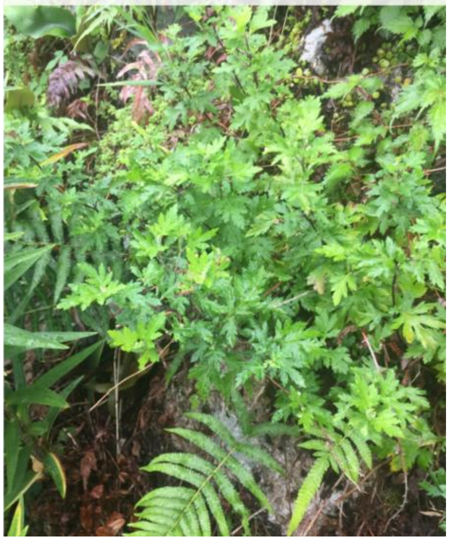

Ko-13

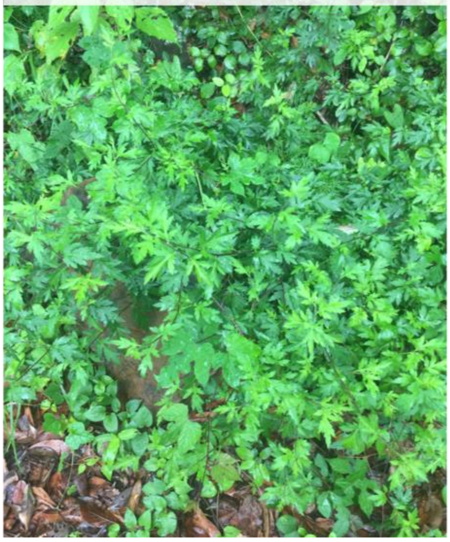

Ko-14

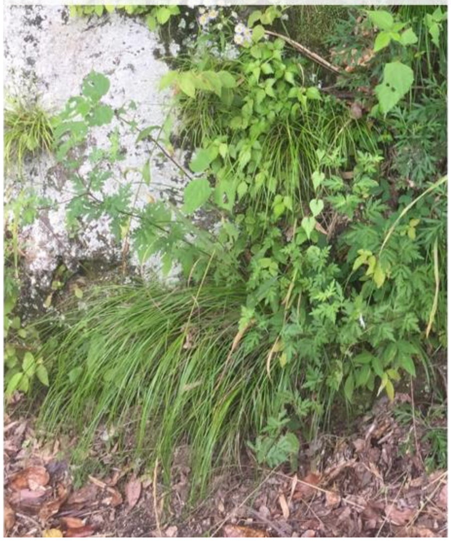

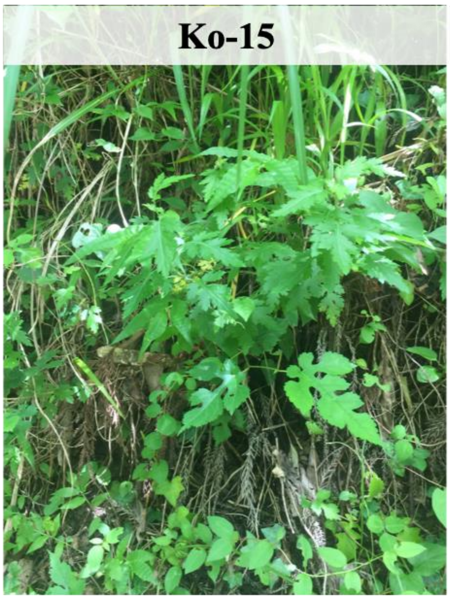
Ko-15

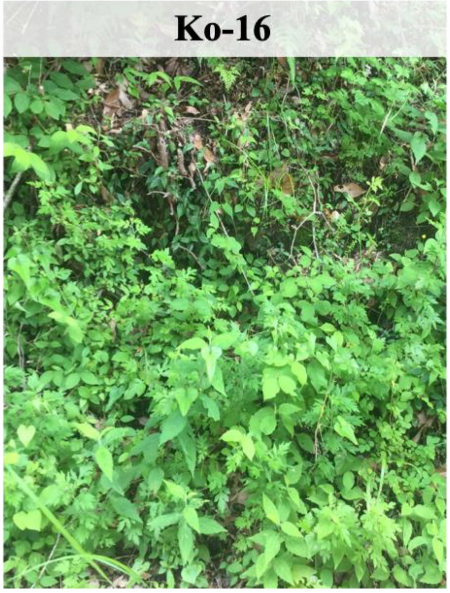
Ko-16

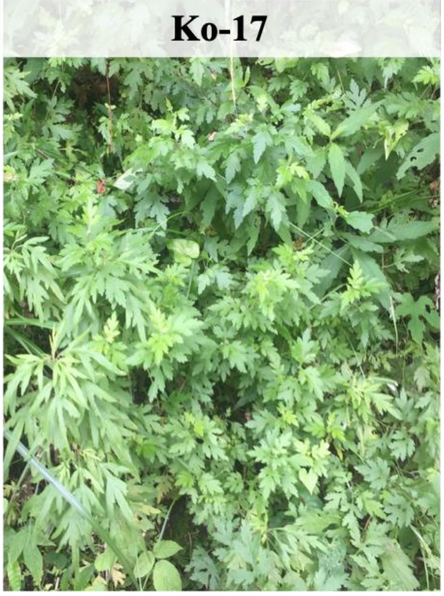
Ko-17

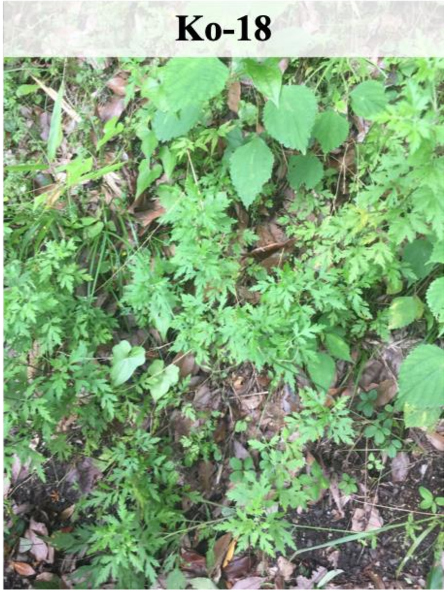
Ko-18

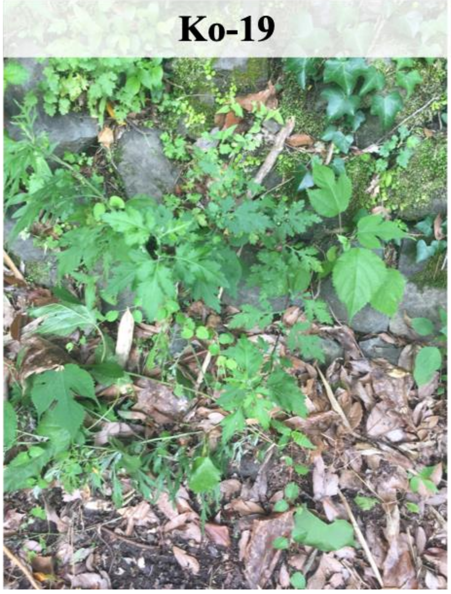
Ko-19

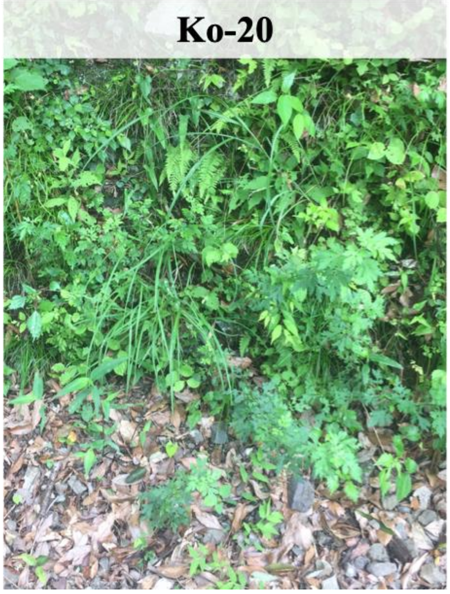
Ko-20

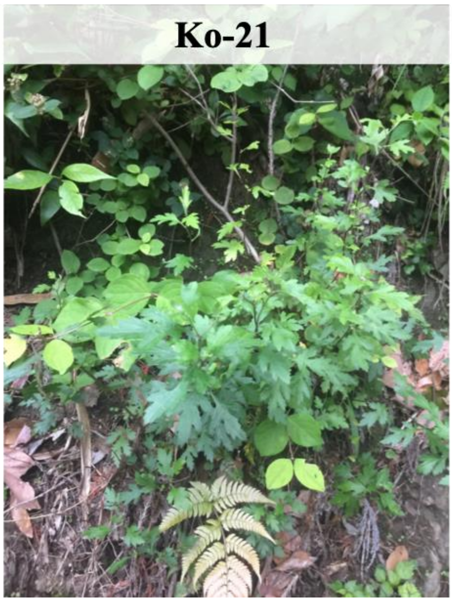
Ko-21

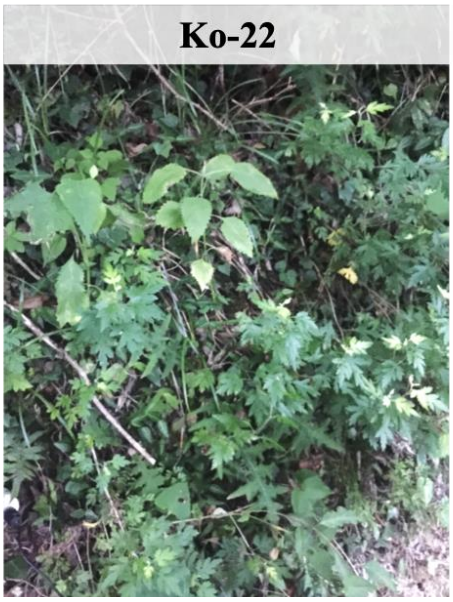
Ko-22

Figure S3. Thematic MaxEnt generated model map of the study area for habitat suitability prediction

Thematic maps of the study area: (**A**) Slope (°), (**B**) Digital Eleveation Model (DEM), (**C**) Aspect (°), (**D**) Curvature, (**E**) Plan Curvature, (**F**) Profile Curvature, (**G**) Hillshade (°), (**H**) Topographic Wetness Index (TWI), (**I**) Elevation (m), (**J**) pH, (**K**) SM150T Output (V), (**L**) Soil Bearing Capacity (t sf^{-1}), (**M**) Electrical Conductivity (mS cm^{-1}), (**N**) Soil Bulk Density (Kg m^{-3}), (**O**) Soil Cation Exchange Capacity (cmolc kg^{-1}), (**P**) Soil Organic Carbon Density (Kg m^{-3}), (**Q**) Soil Organic Carbon Content (g kg^{-1}), (**R**) Soil pH in H_2O, (**S**) Soil Clay Content (%), (**T**) Soil Sand Content (%), (**U**) Soil Silt Content (%), (**V**) Distance to Stream (m), (**W**) Distance to Urban (m), (**X**) Distance to Road (m), (**Y**) Annual Mean Precipitation (mm), (**Z**) Annual Mean Temperature (°)

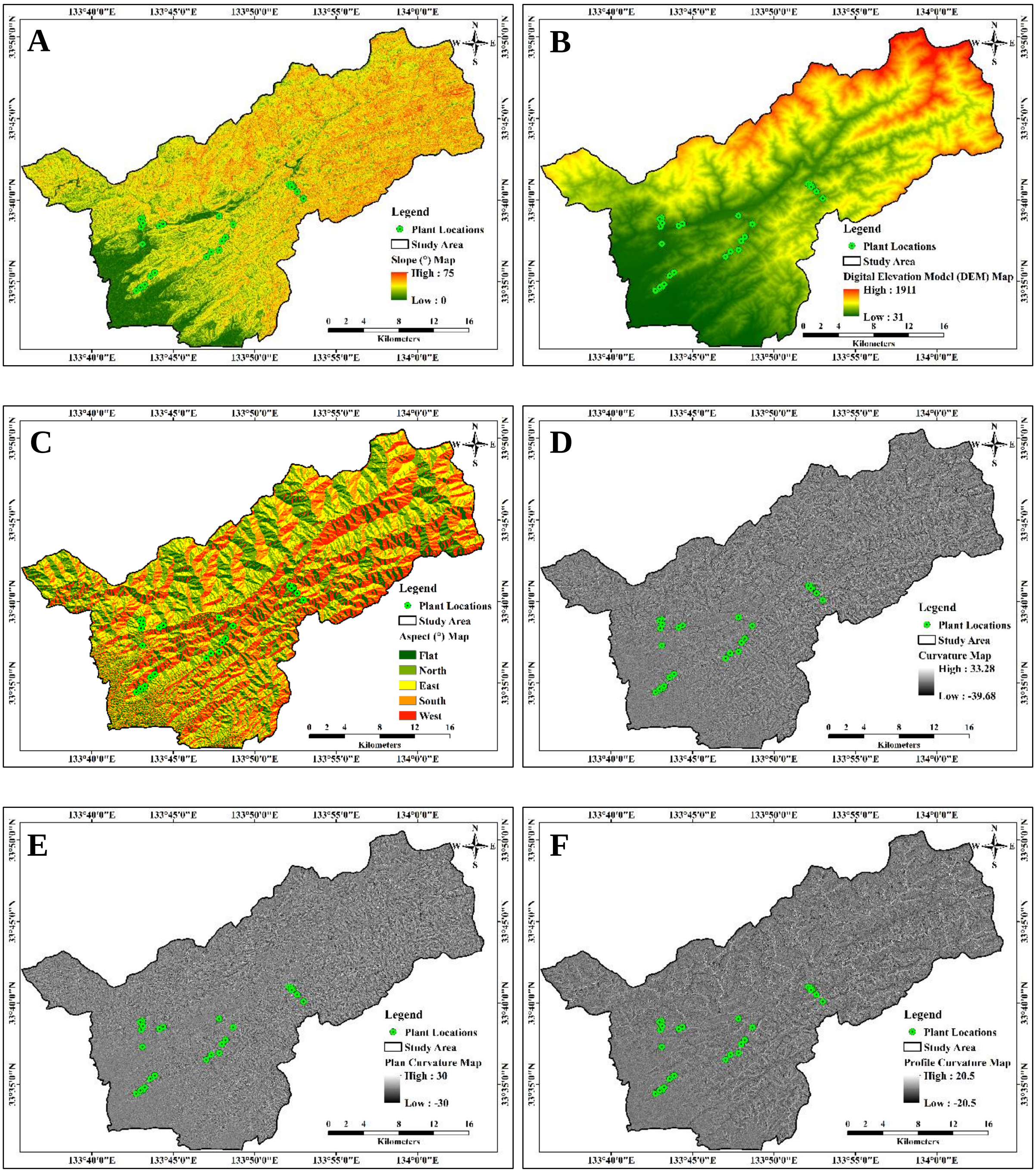

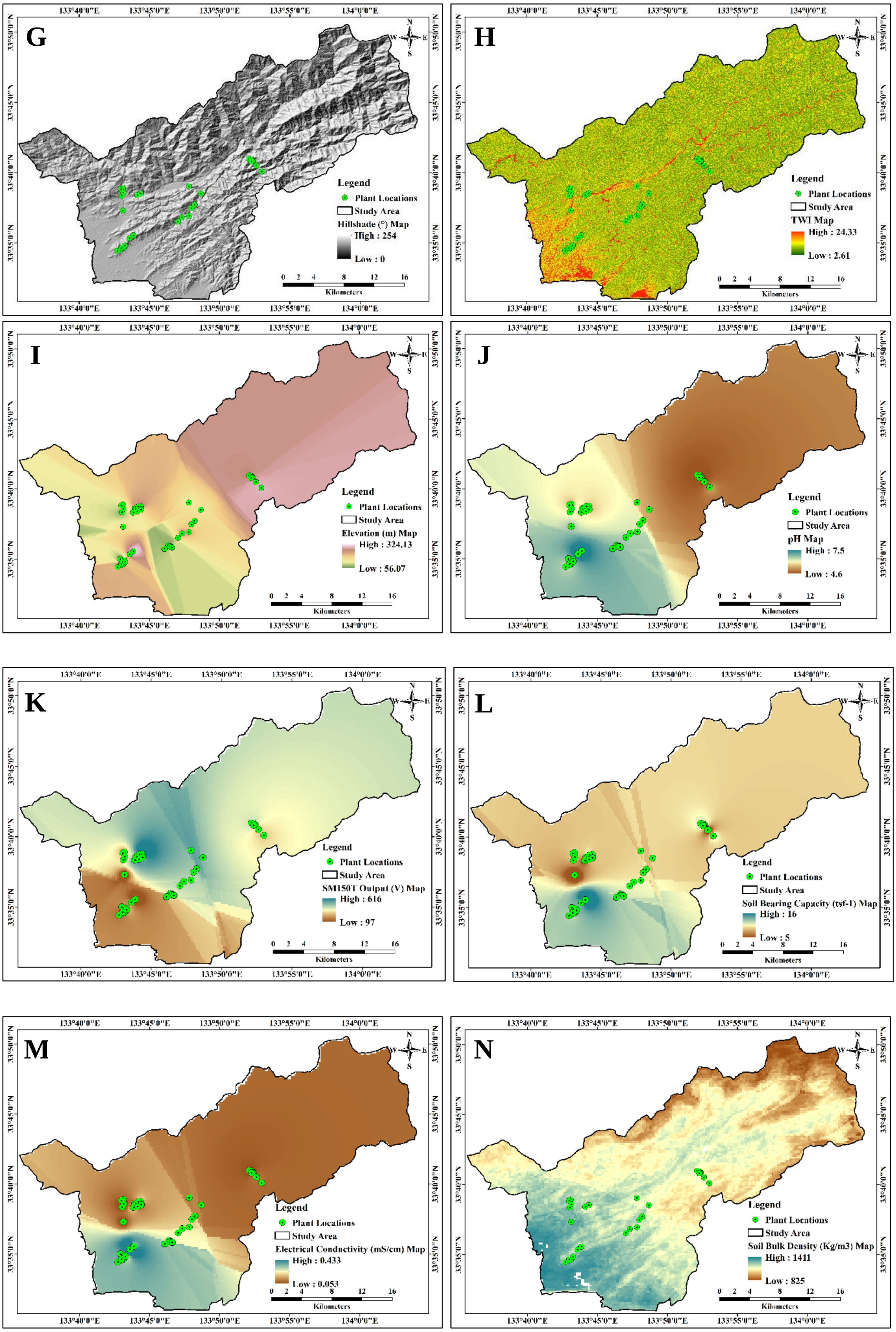

133°40'0"E
133°45'0"E
133°50'0"E
133°55'0"E
134°0'0"E
33°50'0"N
33°45'0"N
33°40'0"N
33°35'0"N
N
W
E
S
0 2 4 8 12 16
Kilometers
G
Legend
Plant Locations
Study Area
Hillshade (°) Map
High : 254
Low : 0
H
Legend
Plant Locations
Study Area
TWI Map
High : 24.33
Low : 2.61
I
Legend
Plant Locations
Study Area
Elevation (m) Map
High : 324.13
Low : 56.07
J
Legend
Plant Locations
Study Area
pH Map
High : 7.5
Low : 4.6
K
Legend
Plant Locations
Study Area
SM150T Output (V) Map
High : 616
Low : 97
L
Legend
Plant Locations
Study Area
Soil Bearing Capacity (tsf-1) Map
High : 16
Low : 5
M
Legend
Plant Locations
Study Area
Electrical Conductivity (mS/cm) Map
High : 0.433
Low : 0.053
N
Legend
Plant Locations
Study Area
Soil Bulk Density (Kg/m3) Map
High : 1411
Low : 825

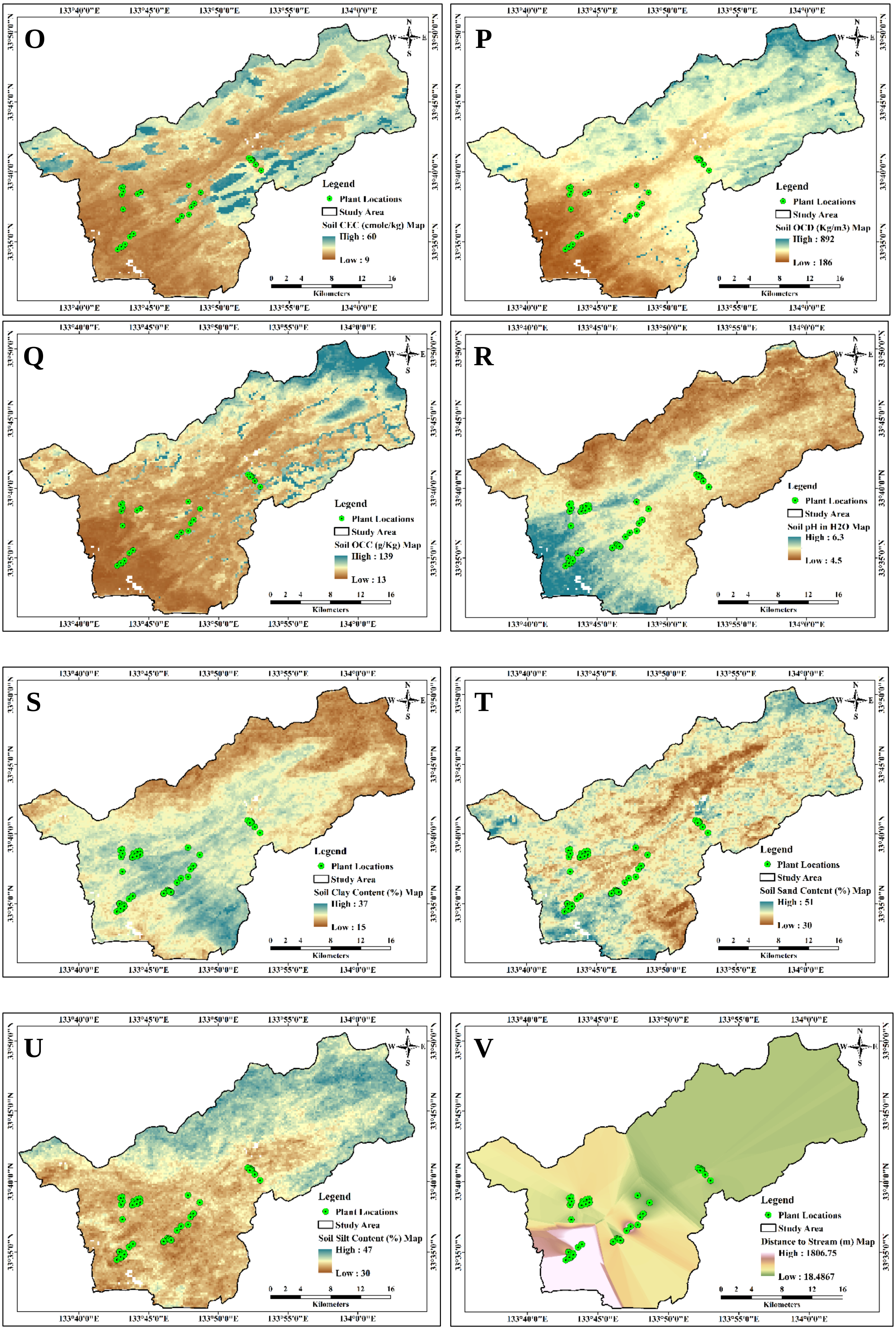
O
Legend
Plant Locations
Study Area
Soil CEC (cmolc/kg) Map
High : 60
Low : 9
P
Legend
Plant Locations
Study Area
Soil OCD (Kg/m3) Map
High : 892
Low : 186
Q
Legend
Plant Locations
Study Area
Soil OCC (g/Kg) Map
High : 139
Low : 13
R
Legend
Plant Locations
Study Area
Soil pH in H2O Map
High : 6.3
Low : 4.5
S
Legend
Plant Locations
Study Area
Soil Clay Content (%) Map
High : 37
Low : 15
T
Legend
Plant Locations
Study Area
Soil Sand Content (%) Map
High : 51
Low : 30
U
Legend
Plant Locations
Study Area
Soil Silt Content (%) Map
High : 47
Low : 30
V
Legend
Plant Locations
Study Area
Distance to Stream (m) Map
High : 1806.75
Low : 18.4867
0 2 4 8 12 16
Kilometers
133°40'0"E
133°45'0"E
133°50'0"E
133°55'0"E
134°0'0"E
33°50'0"N
33°45'0"N
33°40'0"N
33°35'0"N
N
S
E
W

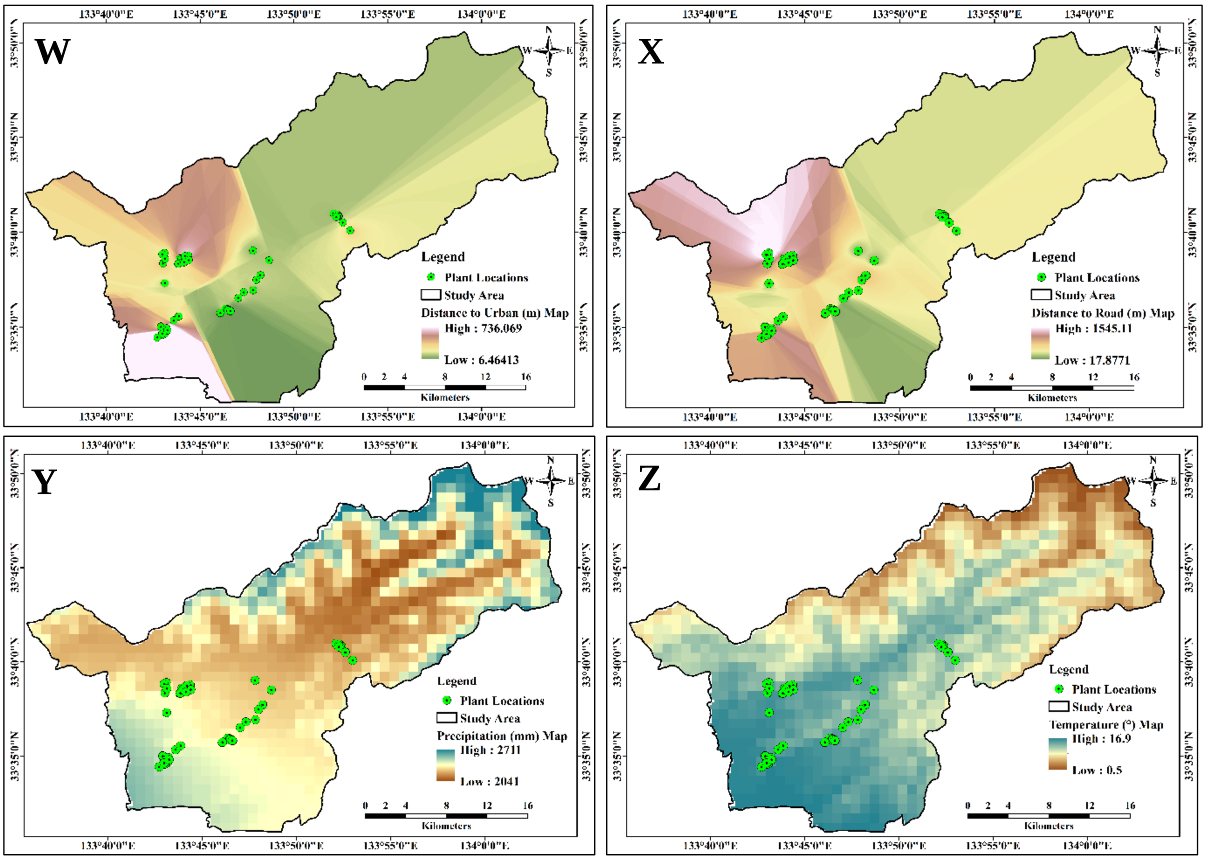

W
Legend
Plant Locations
Study Area
Distance to Urban (m) Map
High : 736.069
Low : 6.46413
Kilometers
X
Legend
Plant Locations
Study Area
Distance to Road (m) Map
High : 1545.11
Low : 17.8771
Kilometers
Y
Legend
Plant Locations
Study Area
Precipitation (mm) Map
High : 2711
Low : 2041
Kilometers
Z
Legend
Plant Locations
Study Area
Temperature (°) Map
High : 16.9
Low : 0.5
Kilometers
